# Gene cassette knock-in in mammalian cells and zygotes by enhanced MMEJ

**DOI:** 10.1186/s12864-016-3331-9

**Published:** 2016-11-28

**Authors:** Tomomi Aida, Shota Nakade, Tetsushi Sakuma, Yayoi Izu, Ayu Oishi, Keiji Mochida, Harumi Ishikubo, Takako Usami, Hidenori Aizawa, Takashi Yamamoto, Kohichi Tanaka

**Affiliations:** 1 Laboratory of Molecular Neuroscience, Medical Research Institute (MRI), Tokyo Medical and Dental University (TMDU), 1-5-45, Yushima, Bunkyo, Tokyo, 113-8510 Japan; 2 Laboratory of Recombinant Animals, MRI, TMDU, 2-3-10, Surugadai, Kanda, Chiyoda, Tokyo, 101-0062 Japan; 3 The Center for Brain Integration Research (CBIR), TMDU, Tokyo, 113-8510 Japan; 4 Department of Animal Risk Management, Chiba Institute of Science, 3 Shiomi-cho, Choshi, Chiba 288-0025 Japan; 5 Department of Mathematical and Life Sciences, Graduate School of Science, Hiroshima University, 1-3-1, Kagamiyama, Higashi-Hiroshima, Hiroshima 739-8526 Japan; 6 Department of Neurobiology, Institute of Biomedical and Health Sciences, Hiroshima University, 1-2-3, Kasumi, Minami-ku, Hiroshima, 734-8553 Japan; 7 Present address: McGovern Institute for Brain Research, Massachusetts Institute of Technology, 43 Vassar St., Cambridge, MA 02139 USA; 8 Present address: Graduate School of Bioagricultural Sciences, Nagoya University, Furo-cho, Chikusa-ku, Nagoya, 464-8601 Japan

**Keywords:** MMEJ, CRISPR/Cas, Gene cassette, Reporter, Flox, Knock-in, Mouse, Exo1, High throughput, Cloning-free

## Abstract

**Background:**

Although CRISPR/Cas enables one-step gene cassette knock-in, assembling targeting vectors containing long homology arms is a laborious process for high-throughput knock-in. We recently developed the CRISPR/Cas-based precise integration into the target chromosome (PITCh) system for a gene cassette knock-in without long homology arms mediated by microhomology-mediated end-joining.

**Results:**

Here, we identified *exonuclease 1* (*Exo1*) as an enhancer for PITCh in human cells. By combining the *Exo1* and PITCh-directed donor vectors, we achieved convenient one-step knock-in of gene cassettes and floxed allele both in human cells and mouse zygotes.

**Conclusions:**

Our results provide a technical platform for high-throughput knock-in.

**Electronic supplementary material:**

The online version of this article (doi:10.1186/s12864-016-3331-9) contains supplementary material, which is available to authorized users.

## Background

Knock-in mice carrying functional gene cassettes have provided invaluable opportunities for in vivo functional analysis of genes and cells in mammalian organisms [1]. Despite the recent rapid displacement of conventional gene-targeting technology in embryonic stem cells [2] by CRISPR/Cas-mediated genome editing [3–5] in mouse zygotes [6–10], the cumbersome task of targeting vector construction containing a gene cassette flanked by long homology arms (generally longer than several kilobases for each) corresponding to every target loci remains unchanged [11, 12]. This limits the feasibility of CRISPR/Cas-mediated, large-scale, high-throughput generation [13, 14] of knock-in mice carrying functional gene cassettes and limits the convenience and availability of the CRISPR/Cas system for mammalian organisms [1, 15].

The homologous recombination (HR)-mediated repair of DNA double-strand breaks (DSBs) induced by CRISPR/Cas has been used to knock-in the gene cassettes in endogenous target genomic loci in mouse zygotes [6, 8, 10]. However, the DSBs are primarily repaired through nonhomologous end joining (NHEJ) in mammalian cells, and thus the frequency of HR-mediated repair is intrinsically low [16]. The efficiencies of HR-mediated gene cassette knock-in in mice are around 10–20%, much less than that (up to 100%) of NHEJ-mediated gene knockout [11, 12]. We recently reported the cloning-free CRISPR/Cas system, which is based on the Cas9 protein and chemically synthesized dual-RNAs (crispr RNA [crRNA] and trans-activating crRNA [tracrRNA]) instead of broadly used single-guide RNA (sgRNA, a chimeric molecule of crRNA and tracrRNA), facilitates HR-mediated gene cassette knock-in in mice by up to 50% [8]. The technological development of CRISPR/Cas-mediated gene cassette knock-in in mice is just beginning, and much improvement is required.

A major alternative pathway for DSB repair, named microhomology-mediated end joining (MMEJ), joins the ends of DSBs by utilizing microhomology for the alignment of broken ends, leading to deletions at the site of the DSBs [17]. Interestingly, the microhomologies were frequently found in the majority of repaired sites of the DSBs induced by CRISPR/Cas in mice [18] and human cells (more than half of the deletions [19]). Taking advantage of the high frequency of MMEJ, we recently developed the highly efficient and convenient CRISPR/Cas or transcription activator-like effector nuclease (TALEN)-based precise integration into the target chromosome (PITCh) system that harnesses MMEJ to knock-in a gene cassette into target genomic loci with extremely short (≤40 bp) microhomologies in cultured cells [20–22], silkworms [20], frogs [20], and zebrafish [23]. In addition to its high efficiency, the PITCh system has the potential to omit the laborious steps of constructing a targeting vector for gene cassette knock-in by the use of microhomologies. The PITCh system may be a key technology for feasible, large-scale, knock-in projects [14] to generate thousands of mice expressing enhanced green fluorescent protein (EGFP) or Cre recombinase under the control of an endogenous promoter, similar to the gene expression nervous system atlas (GENSAT) project for bacterial artificial chromosome transgenic mice [13]; however, its validity in mammalian organisms has not been examined. Here, we show the efficient and highly convenient PITCh-based knock-in strategy in mice by the combination of an MMEJ-directed simplified donor vector, overexpression of the MMEJ-enhancing factor, and the cloning-free CRISPR/Cas system.

## Results

### Generation of knock-in mice carrying a gene cassette by the PITCh system

**Fig. 1:**
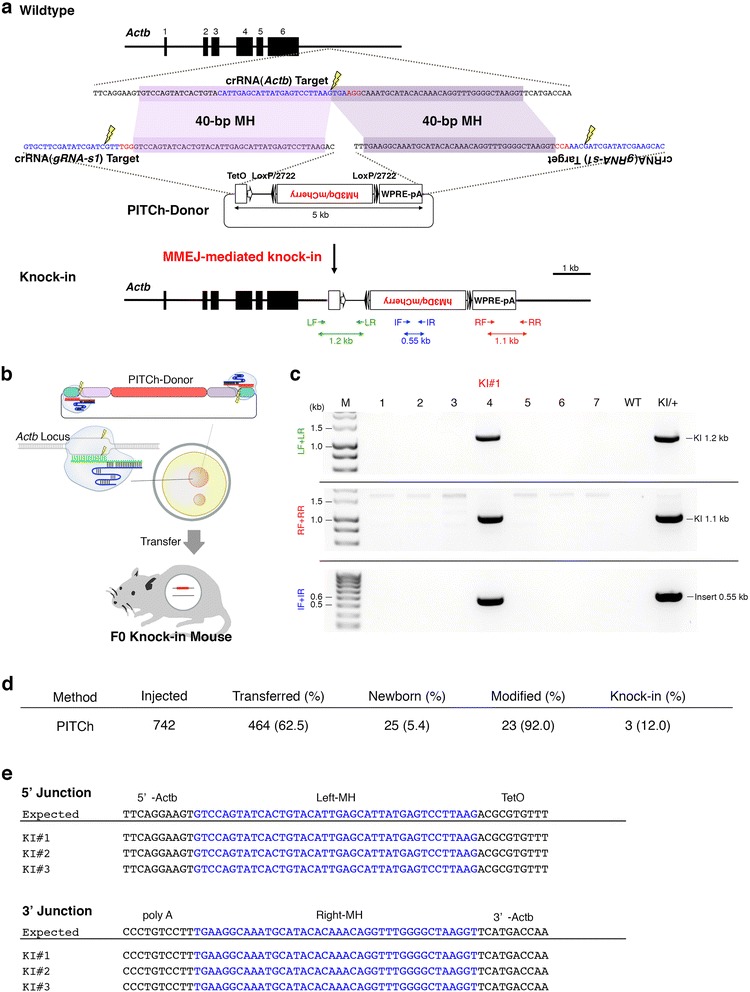
Generation of knock-in mice carrying a gene cassette by the PITCh system. **a** Targeting strategy for the generation of *Actb*-TetO-FLEX-hM3Dq/mCherry knock-in mice by the PITCh system. Purple highlights indicate microhomologies between endogenous *Actb* locus and PITCh-donor. Blue characters indicate CRISPR target sequences. Red characters indicate protospacer adjacent motif (PAM) sequences. Yellow lightnings indicate DSB sites. **b** Schematic diagram of pronuclear injection of Cas9 protein, *Actb* and *gRNA-s1* crRNAs, tracrRNA and PITCh-donor. The red, purple, and blue boxes indicate the insert, *Actb* microhomologies, and *gRNA-s1* target sequences, respectively. **c** PCR screenings of knock-in newborns. **d** Summary of *Actb*-TetO-FLEX-hM3Dq/mCherry knock-in mouse production by the PITCh system. **e** Sequences of boundaries between *Actb* and TetO-FLEX- hM3Dq/mCherry cassette. Blue characters indicate microhomologies. IF: internal forward primer, IR: internal reverse primer, LF: left forward primer, LR: left reverse primer, RF: right forward primer, RR: right reverse primer, MH: microhomology, M: molecular marker, WT: wildtype, KI: knock-in, WPRE: woodchuck hepatitis virus posttranscriptional regulatory element, pA: polyA, and KI/+: tail genomic DNA of F1 heterozygous knock-in pup derived from #13 (KI#2) F0 knock-in mouse

To test the PITCh system in mouse zygotes, we chose the *Actb* locus [24, 25] where we previously knocked-in a 2.5-kb gene cassette by the cloning-free CRISPR/Cas system with a conventional targeting vector containing 2-kb homology arms [8]. In the present study, we knocked-in a 5-kb TetO-FLEX-hM3Dq/mCherry gene cassette (TetO operator [tetO] sequences followed by inverted Gq-coupled human M3 muscarinic DREADD (designer receptors exclusively activated by designer drug, hM3Dq)/mCherry flanked by two pairs of loxP and loxP2722 [FLEX switch]) to the *Actb* locus (Fig. 1a) [1, 8, 15, 26]. Since linearization of the donor is required for gene cassette knock-in by the PITCh system, we designed a synthetic guide RNA sequence (*gRNA-s1*) as the universal guide RNA that shares no sequence homology with the genomic DNA of several mammalian species, including human and mouse [21]. We constructed PITCh-directed donor vectors (PITCh-donor) [21] containing a 5-kb TetO-FLEX-hM3Dq/mCherry cassette flanked by 40-bp left and right microhomologies corresponding to 800 bp downstream of the *Actb* polyA signal and g*RNA-s1* crRNA target sequences (Fig. 1a). The PITCh donor was efficiently digested by in vitro digestion assay (IDA) with *gRNA-s1* crRNA, tracrRNA, and Cas9 protein (Additional file 1: Figure S1).

We then injected the circular PITCh donor together with chemically synthesized *Actb* and *gRNA-s1* crRNAs and tracrRNA, and Cas9 protein into one-cell-stage mouse zygotes (Fig. 1b) [8]. We obtained 25 newborns, and screened their tail genomic DNA by PCR with three different primer sets (Fig. 1a) to identify knock-in mice (Fig. 1c, d and Additional file 1: Figure S2). We found three knock-in mice defined by triple PCR positive carrying a TetO-FLEX-hM3Dq/mCherry cassette at the *Actb* locus (Fig. 1c, d and Additional file 1: Figure S2). Knock-in efficiency was 12% (Fig. 1d). We also found two partial knock-in mice defined by double PCR positive for LF + LR and IF + IR carrying a part of the cassette at the *Actb* locus (mice #10 and #18 in Additional file 1: Figures S2). Next, we sequenced the PCR products of the left and right boundaries between *Actb* and the TetO-FLEX-hM3Dq/mCherry cassette and found the precise knock-in of the cassette we designed (Fig. 1e). Although left boundaries were precisely knocked-in in two partial knock-in mice, we could not determine their right boundaries (data not shown). We also sequenced the PCR products of non-knock-in *Actb* alleles amplified with LF and RR primers. These alleles were modified by NHEJ in 92% of the newborn mice (Fig. 1d and Additional file 1: Figure S3). Collectively, the knock-in mice carrying a gene cassette could be generated by the PITCh system in combination with cloning-free CRISPR/Cas system. However, its efficiency (12%, Fig. 1d) was much lower than that of our previous study (45.5%, [8]), which was accomplished by the combination of a conventional targeting vector with long homology arms and the cloning-free CRISPR/Cas system, although the length of knock-in cassette in this study was larger than that of previous report (5 kb vs. 2.5 kb).

### Genetic screening of MMEJ enhancer

**Fig. 2:**
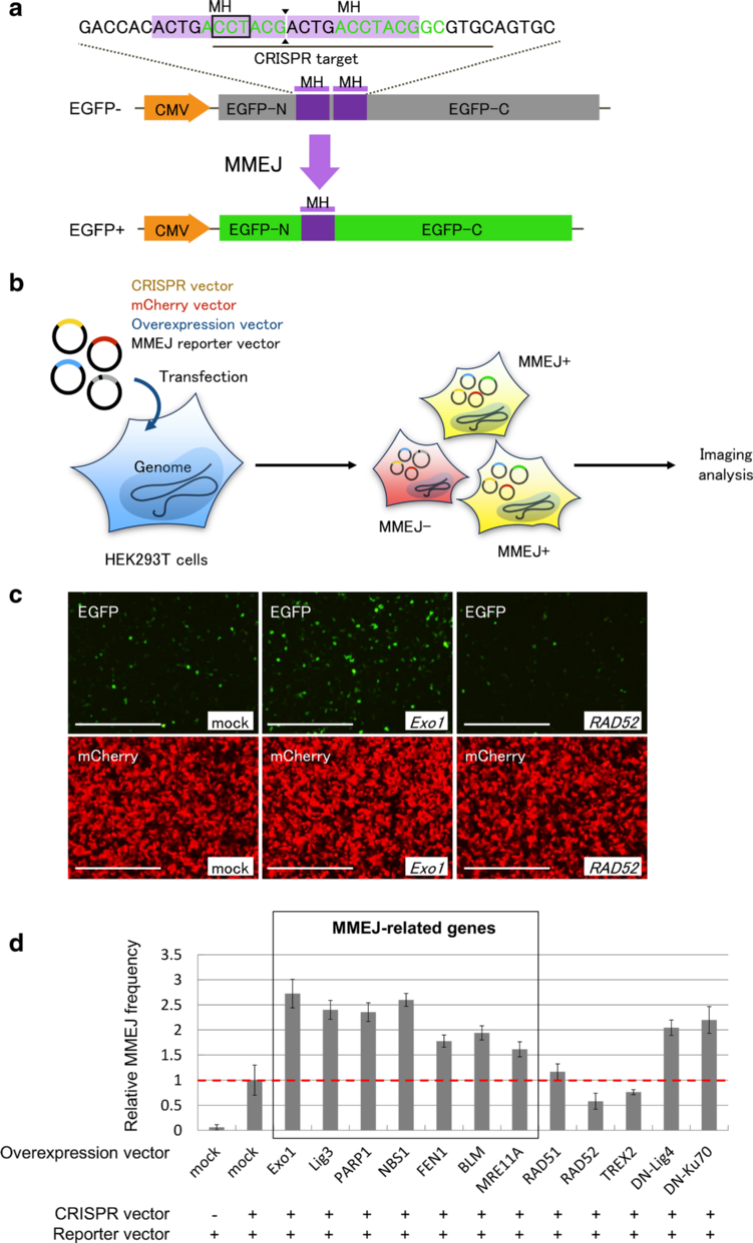
Screening of MMEJ-enhancing factors using a fluorescence reporter assay. **a** Schematic diagram of MMEJ-dependent EGFP recovery. Green letters indicate chromophore sequence. Black line indicates CRISPR target sequence. Black box indicates protospacer adjacent motif (PAM) sequence. Black triangles indicate DSB site. MH, microhomology. CMV, cytomegalovirus promoter. **b** Schematic diagram of HEK293T-based reporter assay, followed by imaging analysis. **c** Fluorescence microscopy images of transfected cells. Bars, 500 μm. **d** Relative frequencies of MMEJ repair, fold to mock overexpression. The MMEJ frequencies were calculated by imaging analysis. Data are expressed as means ± SEM (*n* = 4)

To enhance the efficiency of the MMEJ-mediated gene cassette knock-in, we conducted genetic screening to identify genes that enhance MMEJ. We constructed a fluorescent reporter system to detect MMEJ-mediated repair of DSBs, similar to the previous report [27]. The reporter plasmid expressing inactive (out-of-frame) EGFP was split by a CRISPR target sequence containing two tandem microhomologies under the control of the CMV promoter (Fig. 2a). When the DSBs in the reporter plasmid induced by CRISPR are repaired through MMEJ between two microhomologies, functional in-frame EGFP is reconstituted and EGFP fluorescence is recovered (Fig. 2a). We chose 13 candidate genes from among those involved in DSB repair pathways (*Exonuclease 1* [*Exo1*]*, DNA Ligase 3* [*Lig3*], *Poly (ADP-Ribose) Polymerase 1* [*PARP1*], *Nijmegen Breakage Syndrome protein 1* [*NBS1*], *Flap structure-specific Endonuclease 1* [*FEN1*], *Bloom syndrome RecQ like helicase* [*BLM*], *Meiotic Recombination 11A* [*MRE11A*], *RAD51*, *RAD52, Three Prime Repair Exonuclease 2* [*TREX2*]*,* dominant-negative *DNA ligase 4* [*DN-Lig4*], and dominant-negative *Ku70* [*DN-Ku70*]) and introduced plasmids expressing these cDNAs together with three plasmids; the MMEJ-monitoring reporter, CRISPR/Cas vector targeting the reporter, and mCherry-expressing vector (Fig. 2b) into HEK293T cells. Of these, we found overexpression of MMEJ-related genes and dominant-negative mutants increased the number of EGFP-positive cells (Fig. 2c, d). Especially, MMEJ efficiency by *Exo1* overexpression increased more than 2.5-fold compared to that of the mock overexpression quantified by imaging analysis (Fig. 2d). The overexpression of *TREX2, RAD51*, or *RAD52* did not increase the number of EGFP-positive cells. Conversely, significant reduction of EGFP-positive cells was observed with *RAD52* overexpression compared to that of mock overexpression, consistent with the involvement of this gene in the DSB repair pathway through homologous recombination [28]. These results suggest that overexpression of MMEJ factors, including *Exo1*, and dominant-negative NHEJ factors specifically enhanced MMEJ for DSB repair in human cells. Among these, we focused on *Exo1* in this study, because *Exo1* was reported to increase the mutation frequency of TALEN in mammalian cells and zygotes by potentially acting as an MMEJ enhancer [29].

### *Exo1* enhances PITCh-mediated gene cassette knock-in in human cells

**Fig. 3:**
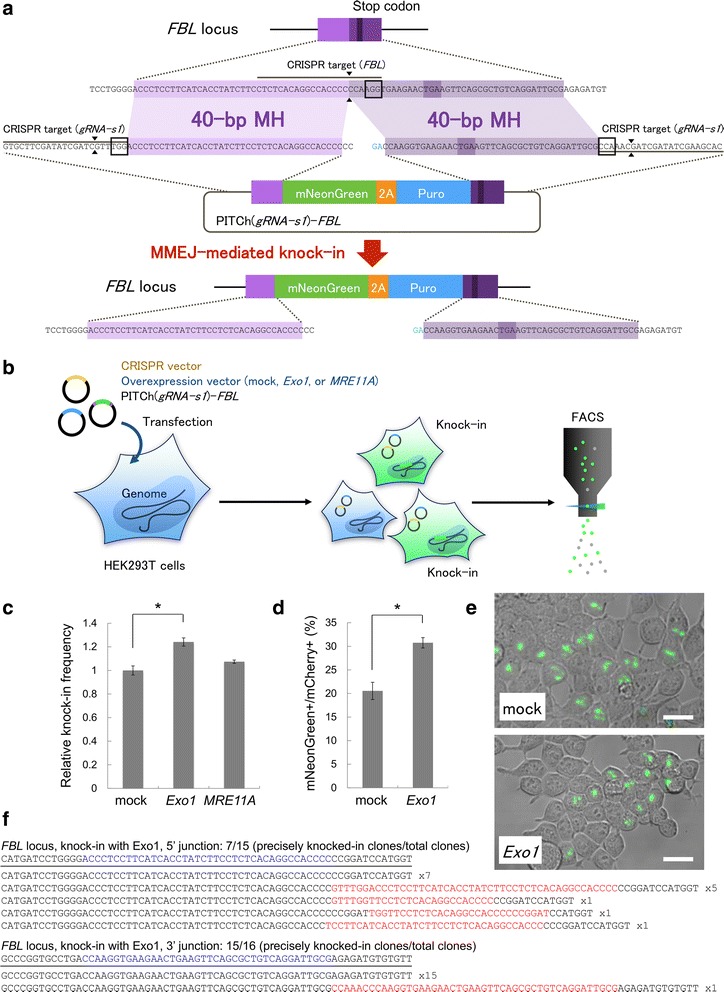
Enhancement of MMEJ-mediated gene knock-in by *Exo1* overexpression at the *FBL* locus in HEK293T cells. **a** Schematic diagram of gene knock-in strategy at the *FBL* locus in HEK293T cells. Black lines indicate CRISPR target sequences. Black boxes indicate PAM sequences. Black triangles indicate DSB sites. MH, microhomology. Puro, puromycin resistance gene. **b** Schematic diagram of gene knock-in in HEK293T cells, followed by FACS analysis. **c** Relative frequencies of gene knock-in, fold to mock overexpression. The knock-in frequencies were calculated by FACS analysis (Additional file 1: Figure S4). Data are expressed as means ± SEM (*n* = 3). Statistical significance was determined by Student’s *t*-test. *P < 0.05. **d** The percentages of mNeonGreen-positive cell areas in mCherry-positive cell areas, calculated by imaging analysis (Additional file 1: Figure S5). Data are expressed as means ± SEM (*n* = 3). Statistical significance was determined by Student’s *t*-test. *P < 0.05. **e** Confocal laser scanning microscopy images of transfected cells. The knocked-in cells showed nucleolar localization of mNeonGreen fluorescence. Bars, 30 μm. **f** DNA sequencing analysis of bacterially cloned PCR products of 5’ and 3’ junctions amplified from the cells knocked-in with *Exo1*. The intended knocked-in sequence is shown at the top of each set of sequences. Blue letters indicate the microhomologies. Red letters indicate insertions

Next, we tested whether *Exo1* enhances the efficiency of PITCh-mediated gene cassette knock-in in an endogenous target locus in human cells. We constructed the PITCh-donor targeting human *fibrillarin* (*FBL*) gene, a component of a nucleolar small nuclear ribonucleoprotein, containing a 1.4-kb mNeonGreen-2A-puromycin cassette [20, 21], although puromycin selection was not used in this study. This cassette was flanked by 40-bp left and right microhomologies corresponding to the last coding exon of the *FBL* and g*RNA-s1* crRNA target sequences (hereafter, PITCh(g*RNA-s1*)-*FBL*) (Fig. 3a). The in-frame C-terminal fusion of *FBL* with the cassette produces the mNeonGreen fluorescence in the nucleolar. We introduced the PITCh(g*RNA-s1*)-*FBL* donor and the all-in-one CRISPR/Cas plasmid [30] expressing two sgRNAs targeting endogenous *FBL* [20] and g*RNA-s1,* and the Cas9 protein, together with plasmids expressing *Exo1* or *MRE11A* cDNAs in HEK293T cells (Fig. 3b). Fluorescence-activated cell sorting (FACS) analysis revealed the knock-in efficiency of the mNeonGreen cassette to the endogenous *FBL* locus with *Exo1* overexpression increased 30% compared to that with mock or *MRE11A* overexpression without drug selection (Fig. 3c and Additional file 1: Figure S4). The knock-in-enhancing effect of *Exo1* was also confirmed by imaging analysis using laser-scanning microscopy (LSM). For imaging analysis, mCherry-expressing vectors were co-transfected with the above-described vectors, and the areas of mNeonGreen- and mCherry-positive cells were calculated (Additional file 1: Figure S5). Consistent with the results of the FACS analysis, *Exo1* increased the percentages of mNeonGreen-positive cells in mCherry-positive cells by 50% (approximately 20 vs. 30% for mock vs. *Exo1* overexpression, Fig. 3d). A magnified view of the LSM images confirmed nucleolar localization of the green fluorescence foci, suggesting that the mNeonGreen-positive cells could be considered as the correctly knocked-in cells (Fig. 3e and Additional file 1: Figure S6). We further confirmed correct gene knock-in in *Exo1*-overexpressed cells by DNA sequencing of 5’ and 3’ junctions (Fig. 3f). Correct knock-in junctions without any mutations were frequently found at both 5’ and 3’ junctions, and their frequencies are comparable with those using a donor with 20-bp microhomology without *Exo1* overexpression reported previously [21] (46.7 and 93.8% with *Exo1*, 80 and 50% without *Exo1*). In addition, we performed Western blotting and toxicity analysis to confirm the effectiveness and safeness of *Exo1* overexpression. The results suggested that protein level of Exo1 was increased by overexpression and it did not affect cell viability (Additional file 1: Figure S7 and S8).

**Fig. 4:**
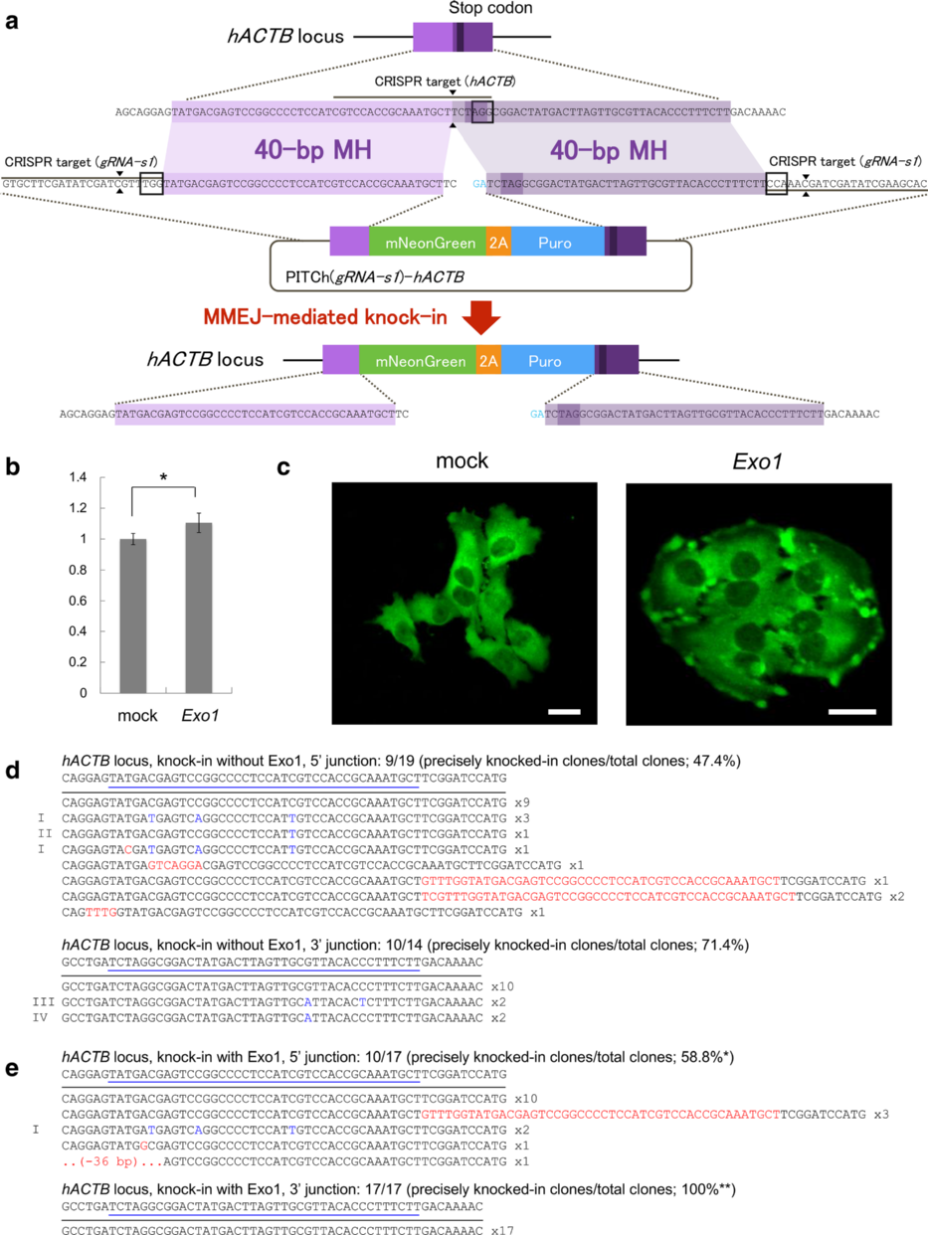
MMEJ-mediated gene knock-in at the *hACTB* locus in HeLa cells. **a** Schematic diagram of gene knock-in strategy at the *hACTB* locus in HeLa cells. Black lines indicate CRISPR target sequences. Black boxes indicate PAM sequences. Black triangles indicate DSB sites. MH, microhomology. Puro, puromycin resistance gene. **b** Relative frequencies of gene knock-in, fold to mock overexpression. The knock-in frequencies were calculated by FACS analysis, similar to Fig. 3b, except that mCherry was used to normalize the transfection efficiency. Data are expressed as means ± SEM (*n* = 3). Statistical significance was determined by Student’s *t*-test. *P < 0.05. **c** Confocal laser scanning microscopy images of transfected cells. The knocked-in cells showed cytoplasmic localization and peripheral accumulation of mNeonGreen fluorescence. Bars, 30 μm. **d**, **e** DNA sequencing analysis of bacterially cloned PCR products of 5’ and 3’ junctions amplified from the cells knocked-in without Exo1 (**d**) and with Exo1 (**e**). The intended knocked-in sequence is shown at the top of each set of sequences. Blue bars indicate the microhomologies. Red letters indicate mutated bases including insertions, deletions, and substitutions. Blue letters indicate polymorphisms found on the off-target genomic sites, related to Fig. 5a. Roman numbers shown at the left side of each set of sequences indicate the allele types, related to Fig. 5a and Additional file 1: Figure S15, S16 and Table S1. Statistical significance was determined by chi-square test. *P < 0.05. ** *P* < 0.01

To confirm the knock-in-enhancing effect of *Exo1*, we knocked-in the same gene cassette at another gene locus in another cell line. We aimed to target human *Actb* (*hACTB*) gene in HeLa cells (Fig. 4a). The knock-in efficiency with *Exo1* overexpression slightly increased compared to that without *Exo1* (Fig. 4b). LSM observation revealed that fluorescent cells showed cytoplasmic localization and peripheral accumulation (Fig. 4c). The correct knock-in was also confirmed by DNA sequencing analysis (Fig. 4d, e). Without *Exo1* overexpression, the percentages of precise knock-in were 47.4 and 71.4% at the 5’ and 3’ junctions, respectively (Fig. 4d). With *Exo1* overexpression, the percentages of precise knock-in were 58.8 and 100% at the 5’ and 3’ junctions, respectively (Fig. 4e). From these direct comparison analysis, it was suggested that *Exo1* overexpression might result in not only higher efficiency but also higher accuracy.

**Fig. 5:**
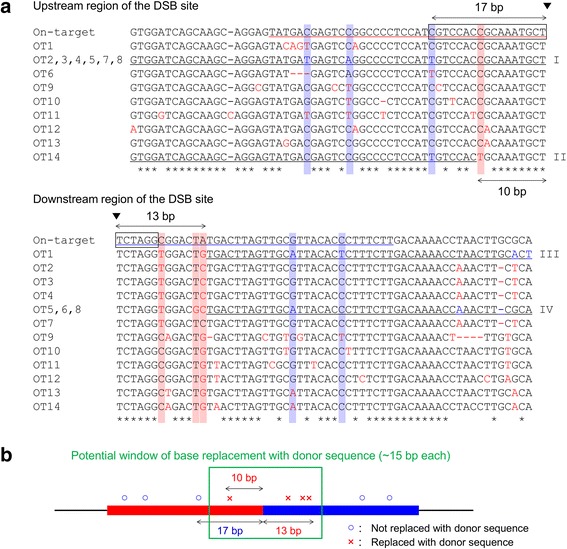
Potential window of MMEJ-dependent base replacement revealed by sequence alignment of on- and off-target knock-ins. **a** Sequence alignment of upstream and downstream regions of the DSB site. OT1–OT14 represents off-target sites, related to Additional file 1: Table S1. Roman numbers shown at the right side of each set of sequences indicate the allele types, related to Fig. 4d, e and Additional file 1: Figure S15, S16 and Table S1. Black triangles indicate the DSB site. Black boxes indicate the CRISPR target sequence. Red and blue underlines indicate the left and right microhomologies, respectively. Black underlines indicate mutated sequences found in sequenced knock-in junctions. Blue letters indicate the polymorphisms found in sequenced knock-in junctions, related to Fig. 4d, e. Red letters indicate the mutations not found in the sequenced knock-in junctions. Asterisks indicate positions with complete base conservation among on- and off-target sites. The positions of bases replaced and not replaced with the donor sequence were masked in red and blue, respectively. The upstream sequences of OT2, OT3, OT4, OT5, OT7, and OT8 and the downstream sequences of OT5, OT6, and OT8 were identical, respectively (Additional file 1: Figure S17). **b** Schematic representation of potential MMEJ window. Red and blue boxes indicate the left and right microhomologies, respectively

It has been well known that functional hACTB protein is produced from a single gene; however, there are many pseudogenes in the human genome [31]. The in silico prediction of the cleavage sites of sgRNA designed to knock-in in *hACTB* locus revealed that there were a lot of candidate sites in the human genome (Additional file 1: Table S1). We thus aligned the upstream and downstream regions of genomic DSB site of the on-target and 14 off-target sites listed according to the scores calculated by the COSMID webtool [32] (Fig. 5a). Compared with the results of sequencing analysis, we found that some base replacements found in the sequenced alleles seemed to be derived from off-target integrants (highlighted in blue letters in Figs. 4d and 5a). More interestingly, mismatched bases near the DSB site were replaced with the donor sequence, whereas those far from the DSB site were not replaced, suggesting that possible window of base replacement via MMEJ repair was determined as around 15 bp each, when the lengths of microhomologies were set as 40 bp (Fig. 5a, b). Collectively, these results suggest that *Exo1* enhances the efficiency, and specificity when targeted multiple genomic sites, of the PITCh-mediated gene cassette knock-in in the endogenous target loci in human cells.

### PITCh-mediated gene cassette knock-in in mice with *Exo1*

**Fig. 6:**
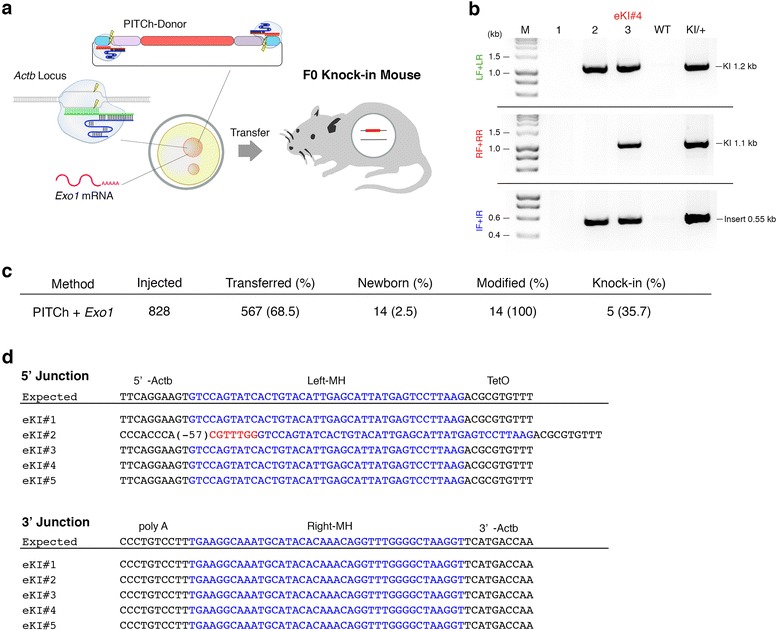
Generation of gene cassette knock-in mice by the enhanced PITCh system. **a** Schematic diagram of pronuclear injection of Cas9 protein, *Actb* and *gRNA-s1* crRNAs, tracrRNA, PITCh-donor, and *Exo1* mRNA. **b** PCR screenings of knock-in newborns. **c** Summary of *Actb*-TetO-FLEX-hM3Dq/mCherry knock-in mouse production by the enhanced PITCh system. **d** Sequences of boundaries between *Actb* and TetO-FLEX- hM3Dq/mCherry cassette. Blue and red characters indicate microhomologies and partial crRNA(*gRNA-s1*) target sequence, respectively. IF: internal forward primer, IR: internal reverse primer, LF: left forward primer, LR: left reverse primer, RF: right forward primer, RR: right reverse primer, MH: microhomology, M: molecular marker, WT: wildtype, KI: knock-in, and KI/+: tail genomic DNA of F1 heterozygous knock-in pup derived from #13 (KI#2) F0 knock-in mouse

We further tested whether *Exo1* enhances the efficiency of PITCh-mediated gene cassette knock-in in the endogenous target locus in mouse zygotes. We injected the circular PITCh donor with chemically synthesized *Actb* and *gRNA-s1* crRNA and tracrRNA and the Cas9 protein with in vitro-transcribed human *Exo1* mRNA into one-cell stage mouse zygotes (Fig. 6a). We obtained 14 newborn mice, of these, five were identified as triple PCR positive knock-in mice carrying a TetO-FLEX-hM3Dq/mCherry cassette at the *Actb* locus (Fig. 6b, c and Additional file 1: Figure S9). We also found one partial knock-in mouse defined by double PCR positive for LF + LR and IF + IR carrying a part of the cassette at the *Actb* locus (mouse #2 in Fig. 6b). Next, we sequenced the PCR products of the left and right boundaries between *Actb* and the TetO-FLEX-hM3Dq/mCherry cassette. We found the precise knock-in of the cassette at both boundaries in four out of five knock-in mice (Fig. 6d). In eKI#2, although the 3’ boundary was precisely knocked-in as we had designed, we found a residual partial sequence of *gRNA-s1* adjacent to the 5’ microhomology of the gene cassette (Fig. 1a) and a 57-bp deletion of the endogenous locus, which did not affect endogenous *Actb* or exogenous cassette functions (Fig. 6d). Although left boundary was precisely knocked-in in one partial knock-in mouse, we could not determine its right boundary (data not shown). The non-knock-in *Actb* alleles were modified by NHEJ in all the newborn mice (Fig. 6c and Additional file 1: Figure S10). Taken together, the knock-in efficiency of the gene cassette by the combination of *Exo1*, PITCh-donor, and cloning-free CRISPR/Cas was 35.7%, with or without functionally negligible mutations (Fig. 6c). These results revealed that increasing tendency of PITCh-mediated gene cassette knock-in by *Exo1* in the endogenous target locus in mouse zygotes.

We further tested off-target cleavage in the knock-in mice generated by the cloning-free CRISPR/Cas system-based PITCh system. We chose five off-target candidate loci containing up to 3 bp mismatches compared to the 20-bp guide sequence of *Actb* crRNA [8]. Among five off-target candidate loci in three knock-in mice without *Exo1* overexpression and five knock-in mice with *Exo1* overexpression, we did not find any off-target digestion (Additional file 1: Figure S11 and Table S2). These results suggest that PITCh-mediated gene cassette knock-in in mouse zygotes by the cloning-free CRISPR/Cas system is highly specific to the on-target locus, and its specificity is not affected by *Exo1* overexpression.

Subsequently, we crossed F0 knock-in mice with a wild-type mouse to investigate germline transmission of the knock-in alleles to the F1 generation. We tested seven knock-in mice, and found successful germline transmission of the knock-in allele in F1 progeny (Additional file 1: Figure S12 and Table S3). The efficiencies of germline transmission were 50% or higher in five out of seven F0 knock-in mice, suggesting that these F0 mice were heterozygous knock-in mice. In contrast, two (KI#2 and eKI#1) showed 33.3% efficiencies of germline transmission. These results suggest that germline mosaicism was occurred in some knock-in mice; however, its frequency was low.

Compared to the newborn rates of our previous work using conventional targeting vector (17%, [8]), it was low in PITCh system (5.4% without *Exo1* and 2.5% with *Exo1*). We hypothesized that linearization of circular PITCh donor may partially explain this. We injected the PCR-amplified linear PITCh donor without vector backbone together with chemically synthesized *Actb* and *gRNA-s1* crRNAs and tracrRNA, and Cas9 protein into one-cell-stage mouse zygotes in the absence of *Exo1* (Additional file 1: Figure S13a), and obtained only one newborn from the transfer of 191 embryos (0.5%, Additional file 1: Figure S13b). Next, we injected PCR-amplified linear PITCh donor at reduced doses, *Actb* crRNA, tracrRNA, and Cas9 protein (Additional file 1: Figure S13a). We obtained one newborn from the transfer of 71 embryos with 5 ng/μl linear PITCh donor (1.4%, Additional file 1: Figure S13b). When we further reduced the dose of linear PITCh donor with 1.5 ng/μl, the newborn rate was improved to 4.2% (5 newborns from 119 embryo transfer) (Additional file 1: Figure S13b). These results suggest that linear donor DNA itself is detrimental to the embryo development, rather than Exo1, and the birth rate can be improved in the donor DNA dose dependent manner.

**Fig. 7:**
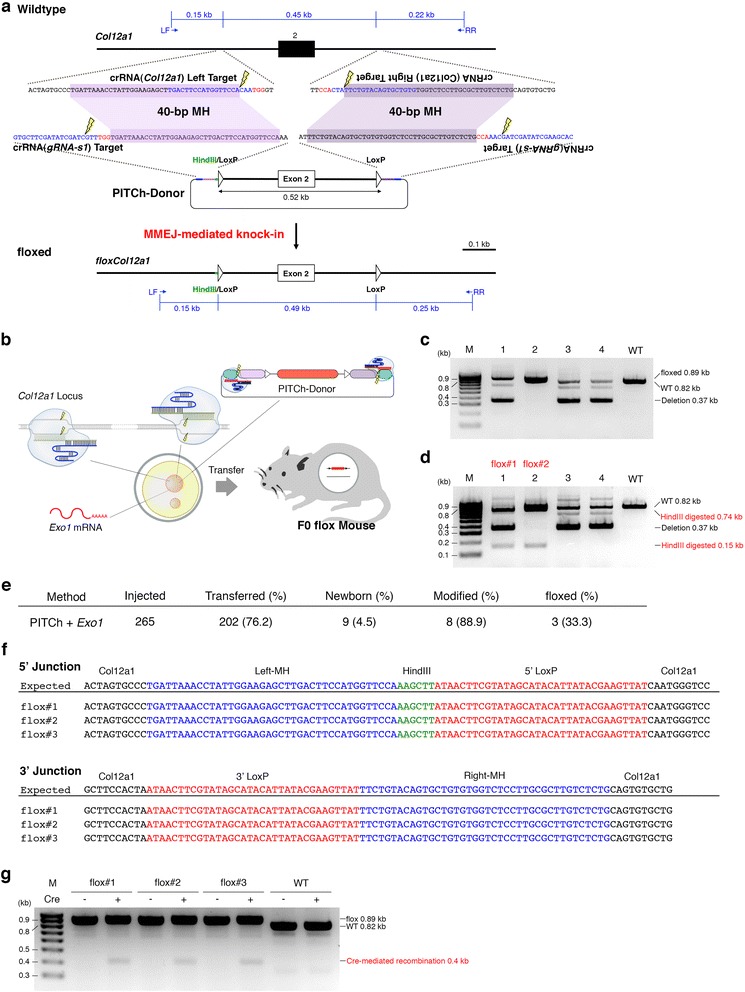
Generation of floxed mice by the enhanced PITCh system. **a** Targeting strategy for the generation of flox*Col12a1* mice by the enhanced PITCh system. Purple highlights indicate microhomologies between endogenous *Col12a1* locus and PITCh-donor. Blue characters indicate CRISPR target sequences. Red characters indicate protospacer adjacent motif (PAM) sequences. Yellow lightnings indicate DSB sites. **b** Schematic diagram of pronuclear injection of Cas9 protein, *Col12a1*-left, -right, and *gRNA-s1* crRNAs, tracrRNA, PITCh-donor, and *Exo1* mRNA. The red, purple, and blue boxes indicate the insert, *Col12a1* microhomologies, and *gRNA-s1* target sequences, respectively. **c** PCR screenings of newborns. **d** PCR-RFLP (restriction fragment length polymorphism) screenings of floxed newborn mice. **e** Summary of flox*Col12a1* mouse production by the enhanced PITCh system. **f** Sequences of boundaries between *Col12a1* and LoxPs. Blue, green, and red characters indicate microhomologies, HindIII sites, and LoxPs, respectively. **g** in vitro Cre-recombination assay. Cloned PCR products of flox alleles from three flox*Col12a1* mice and genomic PCR of wildtype were incubated with or without Cre-recombinase. LF: left forward primer, RR: right reverse primer, MH: microhomology, M: molecular marker, and WT: wildtype

We finally tested whether modified injection protocol with reduced donor DNA concentration could produce knock-in mice by enhanced PITCh system. We chose the *Col12a1* gene and designed a PITCh donor to knock-in the floxed allele for exon 2 substitution as well as a pair of crRNA targeting the flanking introns to excise the target exon (Fig. 7a) because global *Col12a1*-knockout mice showed severe skeletal abnormalities and perinatal lethality [33]. We injected the reduced amount of circular PITCh donor (5 ng/μl) together with chemically synthesized *Col12a1* and *gRNA-s1* crRNAs, tracrRNA, *Exo1* mRNA, and Cas9 protein into one-cell-stage mouse zygotes (Fig. 7b). We screened nine newborns by HindIII digestion of genomic PCR products and sequencing, and identified three as knock-in mice carrying floxed alleles (Fig. 7c–e, and Additional file 1: Figure S14). Further, we cloned and sequenced the PCR products, and found the precise knock-in of a HindIII site and two LoxP sites on the same alleles in all three knock-in mice as we designed (Fig. 7f). The precise integration of two LoxP sites on the same allele was also confirmed by excision of the allele by Cre recombinase (Fig. 7g), although Col12a1 protein level was not investigated. The knock-in efficiency of floxed *Col12a1* was 33.3% (Fig. 7e) comparable to that of *Actb* by enhanced PITCh system (Fig. 6c). Collectively, the modified injection protocol is enough to produce the knock-in mice carrying a floxed allele by enhanced PITCh system with sufficient efficiency.

## Discussion

Although we previously developed an improved CRISPR/Cas system for HR-mediated gene cassette knock-in in mice by using chemically synthesized dual-RNAs and commercially available Cas9 protein [8], constructing targeting vectors containing long homology arms corresponding to every target locus has remained cumbersome and fundamentally unchanged for 30 years [2]. In this report, we applied the PITCh system for the generation of knock-in mice, and this resulted in the omission of the laborious steps of constructing targeting vectors. Our new method has major advantages for researchers generating gene cassette knock-in mice.

First, our method enables extremely convenient and high-throughput donor vector production. Because the MMEJ-directed donor vector can easily be prepared by a single PCR and TA-cloning using primers conjugated with 40-bp microhomology and g*RNA-s1* crRNA target sequence from any template plasmid containing various gene cassettes (EGFP, Cre, and any functional gene cassettes) [21], the long homology arms, which have to be amplified from genomic DNA, are no longer required. Thus, for example, nearly 100 MMEJ-directed donor vectors corresponding to nearly 100 target loci can be prepared by a single PCR reaction in a 96-well plate and those TA-cloning, enabling a large-scale, high-throughput, knock-in mouse project [13, 14]. By combining the enhanced PITCh system with the cloning-free CRISPR/Cas system, critical steps are to design primers to PCR amplify various gene cassettes for 40-bp microhomology arms and their subsequent cloning. Thus, our new method provides a novel approach to a knock-in gene cassette in mice on scales of both a small laboratory and a large international consortium.

Second, the enhanced PITCh system enabled gene cassette knock-in mouse generation. We successfully generated knock-in mice carrying a 5-kb gene cassette by the original PITCh system [20, 21] in combination with the cloning-free CRISPR/Cas system with about 10% efficiency. Its efficiency was acceptable and comparable to previous reports using an HR-based conventional targeting vector with long homology arms [6, 10]. We performed genetic screening to identify an MMEJ enhancer and found that the co-delivery of *Exo1* enhanced knock-in efficiency in human cells. We showed that the knock-in efficiency of a 5-kb gene cassette in mice was 30% by the combination of *Exo1*, the PITCh system, and the cloning-free CRISPR/Cas system. We further showed the validity of the enhanced PITCh system in mice in another genomic locus by knocking in floxed alleles. These efficiencies are sufficient for conventional use, and thus, several knock-in newborn mice can be obtained from small-scale embryonic manipulation, leading to flexible, high-throughput and high-variety, low-volume mouse genetics.

Despite the use of short microhomology (40 bp) in the PITCh system, the junctions between the endogenous locus and the insert were precisely integrated in the majority of the knock-in mice (21 out of 22 boundaries from 11 knock-in mice). We also found a short deletion of the endogenous locus at the 5’ junction in one knock-in mouse treated with *Exo1*, suggesting NHEJ-mediated repair occurred between the deleted genome and the digested donor end. Although the imprecise repair around the targeted integration locus did not affect endogenous gene or exogenous cassette functions when the introns or intergenic regions were targeted, further technological improvements are required for precise targeting of the coding regions to fuse the gene cassettes with endogenous gene products. In addition, we found a few partial knock-in mice defined by double PCR positive for LF + LR and IF + IR (e.g. mice #10 and #18 in Additional file 1: Figures S2 and mouse #2 in Fig. 6b) in the presence or absence of *Exo1*. The whole plasmid integration by insufficient digestion of right boundary of the PITCh-donor or deletion of primer-binding site in mouse genome or PITCh-donor caused by DNA end resection might result in failure of right boundary amplification. Nevertheless, the enhanced PITCh system can generate knock-in mice with sufficient efficiency, and the majority of these mice had precise integration of the gene cassette to the endogenous locus, providing opportunities to select precisely targeted knock-in mice.

We found *Exo1* as an MMEJ enhancer both in human cells and mouse zygotes. *Exo1* is a major 5’–3’ exonuclease, which is critical for the end resection of DSBs to promote MMEJ [17]. The mutation and overexpression of *Exo1* in yeasts are reported to reduce and increase MMEJ, respectively [34, 35]. Consistent with this view, we recently reported that the overexpression of *Exo1* increased knockout efficiency possibly through increased MMEJ in rat zygotes [29]. Thus, in the enhanced PITCh system, *Exo1* may act as the MMEJ enhancer by resection of the DSB ends of both the endogenous locus and the donor vector, promoting their alignment using microhomology. We also showed that various MMEJ-related genes and dominant-negative mutants of NHEJ factors enabled to enhance MMEJ. The enhancer effect of these genes in gene knock-in should be examined in the future study. On the other hand, additional three genes (*TREX2, RAD51*, and *RAD52*) involved in the repair pathway of DSBs did not enhance MMEJ. *TREX2* is also a DNA end-processing enzyme, which reportedly enhances gene disruption when overexpressed with site-specific nucleases [36]. However, it slightly decreased MMEJ frequency, suggesting that it is involved in other DSB repair pathways rather than in the MMEJ pathway. Similarly, *RAD51* and *RAD52*, known as HR-specific factors [28], also decreased the MMEJ frequency.

We additionally investigated whether mismatched bases in microhomologies can be replaced with the donor sequence during the PITCh knock-in, by simultaneously targeting the *hACTB* locus and other pseudogenes. Although there might be an amplification bias of 5’ and 3’ junctions depending on the sequence variations between primers and target sites (Additional file 1: Figure S15), we found two types of off-target knock-in alleles in 5’ junction and another two types in 3’ junction. The sequence comparison between genomic sequences and sequenced alleles revealed that the base-replacing window was around 15 bp. This parameter might be changed when the different length of microhomology is used or different site-specific nucleases such as TALENs are used, but this finding would be important to design both the target site and microhomologies for PITCh knock-in, especially when multiple genomic loci are simultaneously targeted.

The injection of linear PCR donors was enough to reduce the newborn mouse rates in a dose dependent manner. Consistent with our data, Miura et al. also reported that injection of linear double strand DNA donor strongly reduce the newborn mouse rates [37]. These results suggest that the low birth rate of newborn mice in PITCh system is mainly caused by toxicity of linear donor DNA generated by CRISPR-mediated in situ digestion. The survival rate can be improved by the reduction of PITCh donor dose. We showed this modified protocol could also produce knock-in mice without reduction of its efficiency. Although transfection of the various doses of *Exo1* expressing plasmids to HEK293T cell did not affected their survival, its overexpression in mouse zygotes reduced the newborn rate. A recent report in the human cell line showed that overexpression of TREX2, an end-processing enzyme, increased both on- and off-target mutation rates [38], whereas other showed opposite results [36]. This suggests the possibility that the overexpression of *Exo1* may also increase off-target mutation rate, leading to the reduction of newborn mouse rate, and thus, the careful guide RNA design is also essential.

The development of small molecules that activate *Exo1* may be useful for improvement of knock-in efficiency. Scr7, a small compound inhibitor of DNA ligase IV, which is responsible for NHEJ, was recently developed [39] and harnessed to improve the efficiency of HR-mediated knock-in of single nucleotide substitutions and small tags in mice [12, 40] and human cells [41] by suppressing NHEJ. Further, the two small compounds (L755507 and Brefeldin A) were also identified as enhancers for HR by chemical screening using the fluorescent HR reporter system [42]. Similarly, RS-1, a stimulator of *Rad51* [43], is shown to enhance knock-in of long inserts in cellular knock-in screening [44] and rabbits [45]. Thus, by utilizing the fluorescent reporter system to detect MMEJ-mediated repair of DSBs, which we used herein, the small compounds that enhance MMEJ and PITCh-mediated gene cassette knock-in could be identified.

## Conclusions

We developed a convenient HR-independent method for one-step generation of knock-in human cells and mice carrying gene cassettes and floxed allele without long homology arms by enhanced PITCh system.

## Methods

### Plasmid construction for human cell experiments

The CRISPR/Cas9 vector expressing Cas9 nuclease and an sgRNA targeting EGFP reporter vector was constructed using pX330 vector (Addgene Plasmid #42230) according to the previously described protocol [46]. The all-in-one CRISPR/Cas9 vector expressing Cas9 nuclease and two sgRNAs targeting the *FBL* or *hACTB* gene and the donor vector was constructed using the Multiplex CRISPR/Cas9 Assembly System Kit (Addgene Kit #1000000055) as described previously [30]. The oligonucleotide sequences for sgRNA templates were listed in Additional file 1: Table S4.

The overexpression vectors were constructed using an RT-PCR and In-Fusion HD Cloning Kit. Briefly, the full coding sequences for the 14 genes listed in Fig. 2d were amplified using an RT-PCR from total RNAs extracted from HEK293T cells. The amplified cDNAs were cloned into ptCMV-136/63-VR-NG vector (Addgene Plasmid #50700) [47], replacing the transcription activator-like effector nuclease-coding sequence with each cDNA. The sequences of primers used to construct the overexpression vectors were listed in Additional file 1: Table S4.

The EGFP reporter vector for monitoring MMEJ frequency was constructed using a PCR and In-Fusion HD Cloning Kit (Takara). PITCh(*gRNA-s1*)-*FBL* and PITCh(*gRNA-s1*)-*hACTB* donor plasmids were constructed using a PCR and TA-cloning with DynaExpress TA PCR Cloning Kit (pTAC-2) (BioDynamics Laboratory Inc.) or TArget Clone -Plus- (Toyobo). The full plasmid sequences of EGFP reporter vector, PITCh(*gRNA-s1*)-*FBL* donor vector, and PITCh(*gRNA-s1*)-*hACTB* donor vector are shown in Additional file 1: Figure S17.

### Cell culture and transfection

HEK293T and HeLa cells were maintained in Dulbecco’s modified Eagle’s medium supplemented with 10% fetal bovine serum. Lipofectamine LTX (Life Technologies) and Opti-MEM (Life Technologies) were used to transfect plasmids, according to the manufacturer’s instructions. Plasmid concentrations, cell numbers, and dishes used were as follows: for the MMEJ-monitoring reporter assay, 100 ng each for all the plasmid vectors (EGFP reporter vector, CRISPR/Cas vector, mCherry vector, and each overexpression vector) into 6 × 10^4^ HEK293T cells using a 96-well plate; for the FACS and DNA sequencing analyses and the imaging quantification using LSM of *FBL* knocked-in cells, 100 ng each for all the plasmid vectors (PITCh donor vector, CRISPR/Cas vector, and mock, *Exo1*, or *MRE11A* vector with or without mCherry vector) into 5.0 × 10^4^ HEK293T cells using a 12-well plate; for the fluorescence observation of *FBL* knocked-in cells, 50 ng each for all the plasmid vectors (PITCh donor vector, CRISPR/Cas vector, mock or *Exo1* vector, and mCherry vector) into 3 × 10^4^ HEK293T cells using a 96-well plate; for gene knock-in at the *FBL* locus and co-transfection of *FBL-mCherry* fusion gene, 100 ng each for all the plasmid vectors (PITCh donor vector, CRISPR/Cas vector, and *FBL-mCherry* vector) into 2.5 × 10^4^ HEK293T cells using a 12-well plate; for the FACS analysis of *hACTB* knocked-in cells, 100 ng each for all the plasmid vectors (PITCh donor vector, CRISPR/Cas vector, mCherry vector, mock or *Exo1* vector, and the vector expressing puromycin resistance gene) into 2 × 10^5^ HeLa cells using a 12-well plate; for the DNA sequencing and fluorescence observation of *hACTB* knocked-in cells, 8.3–50 ng each for all the plasmid vectors (PITCh donor vector, CRISPR/Cas vector, mCherry vector, mock or *Exo1* vector) into 3 × 10^4^ HeLa cells using a 96-well plate; for Western blotting analysis, 2 μg for mock or *Exo1* vector into 5 × 10^4^ HEK293T cells using a 6-well plate; for toxicity analysis, 1, 3.5, 12.2, 42.9, and 150 ng each for mock or *Exo1* vectors, or 150 ng for zinc-finger nuclease (ZFN) vector, pSTL-ZFA36 [48], and 150 ng for mCherry vector into 6 × 10^4^ HEK293T cells using a 96-well plate. After transfection, cells were cultured in the growth medium described above for 1 day (reporter assays), 3 days (knock-in experiments and Western blotting), or 2 or 5 days (toxicity analysis), with or without transferring the cells to a larger culture plate. For the FACS analysis of *hACTB* knocked-in cells, puromycin selection was conducted during 24–72 h post-transfection.

### FACS analysis

Cells were collected, suspended in PBS, and filtered with a Flowmi Tip Strainer (Bel-Art Products). The number of cells with green (mNeonGreen) and red (mCherry) fluorescence, where needed, was counted using a BD FACS Calibur 4A (BD Biosciences) with a 488-nm laser and the corresponding fluorescence filters. A total of 10,000 cells were recorded for each sample. For toxicity analysis, cell survival rates were determined as the percentages of the number of mCherry-positive cells in total cell counts, as described previously [49].

### Fluorescence microscopy

For the reporter assays, fluorescence was observed and cell images were captured using a fluorescence microscope (Olympus CKX41) directly in the cultured plates. For the knock-in experiments, cells were moved to collagen-coated glass-bottom 24-well plates at 72 h post-transfection, cultured for additional 24 h, and fixed with 4% paraformaldehyde in PBS. Fluorescence was observed and cell images were captured with a 473-nm and 594-nm lasers using a confocal laser-scanning microscope (Olympus FV-1000D).

### Imaging analysis using the ImageJ software

For the reporter assays, the areas containing red fluorescence (mCherry) and green fluorescence (EGFP) were calculated from the captured images using the ImageJ software (https://imagej.nih.gov/ij/). The MMEJ efficiency was determined as the percentages of the EGFP-positive areas in the mCherry-positive areas. For the knock-in experiments, imaging analysis was performed as described in Additional file 1: Figure S4.

### Sequencing analysis for human cell experiments

The 5’ and 3’ knock-in junctions of *FBL* and *hACTB* genes were amplified by PCR using the primers listed in Additional file 1: Table S4 from the cells collected at 72 h post-transfection without any antibiotic or fluorescence selection. Subsequently, the PCR products were bacterially cloned using the TA-cloning method and sequenced.

### Western blotting

Cells were collected and dissolved in sample buffer (final concentrations: 0.125 M Tris-HCl, pH 6.8, 4% SDS, 20% glycerol, 10% β-mercaptoethanol, 0.005% bromophenol blue). Western blotting was performed as previously described [50]. Briefly, after denaturation by heating at 95 degree for 10 min, 10 μg of each sample was separated by 4–20% SDS-PAGE (Bio-Rad) along with a protein standard ladder (Nippon Genetics). The samples were then transferred to PVDF membranes. The membranes were cut and blocked in TBS with 0.1% Tween 20 (TBS-T) and 5% skim milk for 1 h at room temperature, then incubated with primary monoclonal antibodies against Exo1 (1:100, Thermo Scientific, Ab-4, clone 266) and β-Actin (1:1000, Santa Cruz Biotechnology, sc-47778) in TBS-T containing 1% skim milk at 4° overnight. Next, the membranes were washed, incubated with HRP-conjugated anti-mouse IgG secondary antibody (1:5000, Jackson ImmunoResearch Laboratories, 715-035-151) at room temperature for 1 h, washed, and visualized with Luminata Forte Western HRP substrate (Millipore). Gel images were taken every 1 min for 20 min using Image Lab software (Bio-Rad). Band intensities of gel images within linear signal range were quantified using Image Lab software and normalized with band intensities of β-Actin.

### Animal experiments

All research and animal care procedures were approved by the Tokyo Medical and Dental University Animal Care and Use Committee. Mice were housed in groups of 3-5 animals per cage and maintained on a regular 12 h light/dark cycle (8:00–20:00 light period) at a constant 25 °C. Food and water were available *ad libitum*.

### Targeting vector for mouse experiments

*Actb*-TetO-FLEX-hM3Dq/mCherry PITCh-donor: TetO-FLEX-hM3Dq/mCherry cassette was PCR-amplified from an original plasmid with PrimeSTAR GXL DNA Polymerase (Takara) and primers conjugated with *Actb* microhomologies and g*RNA-s1* crRNA target sequences (Fig. 1a), or with *Actb* microhomologies (Additional file 1: Figure S13). PCR products were directly used as linear PITCh donors (Additional file 1: Figure S13). Then, PCR products were cloned into plasmids using Mighty TA-cloning Kit (Takara). The PCR products were also inserted into plasmids using In-Fusion HD Cloning Kit (Takara), in some cases.

flox*Col12a1* PITCh-donor: The genomic region containing exon 2 of *Col12a1* was PCR-amplified as described above with primers conjugated with LoxPs. Then, second PCR was performed with primers conjugated with *Col12a1* microhomologies and g*RNA-s1* crRNA target sequences. Then, PCR products were cloned into plasmids using Mighty TA-cloning Kit (Takara).

### in vitro mRNA transcription

*Exo1* mRNA was in vitro transcribed using mMESSAGE mMACHINE T7 ULTRA Kit (Life Technologies). *Exo1* mRNA was purified with MEGAclear Kit (Life Technologies) and eluted with Nuclease-free water (Life Technologies). The quality of mRNA was analyzed by NanoDrop (Thermo Scientific) and Bioanalyzer (Agilent Technologies).

### Cas9 proteins

The recombinant Cas9 proteins were obtained from Fasmac, New England BioLabs (NEB), and PNA Bio.

### Chemical synthesis of crRNA and tracrRNA

crRNAs and tracrRNA (Additional file 1: Table S4) were chemically synthesized and purified by high pressure liquid chromatography (Fasmac).

### in vitro digestion assay

IDAs were performed as previously described [8] with 100 ng of the PITCh-donor vector, g*RNA-s1* crRNA, tracrRNA and the Cas9 protein.

### Injection

Injections were performed as previously described [8]. For *Actb*-TetO-FLEX-hM3Dq/mCherry knock-in mice, Cas9 proteins, *Actb* and *gRNA-s1* crRNAs, tracrRNA, and PITCh-donor vectors with or without *Exo1* mRNA (30 ng/μl, 0.61 pmol/μl, 10 ng/μl, and 10 ng/μl, respectively) were injected into pronuclei of one-cell stage BDF1 zygotes. For linear PCR donor injection, Cas9 proteins, *Actb* crRNA (and *gRNA-s1* crRNA), tracrRNA, and linear PCR PITCh-donor (1.5, 5, or 10 ng/μl) were injected. For flox*Col12a1* mice, Cas9 proteins, left and right *Col12a1* crRNAs, tracrRNA, and PITCh-donor vectors with *Exo1* mRNA (30 ng/μl, 0.61 pmol/μl, 5 ng/μl, and 10 ng/μl, respectively) were injected into pronuclei of one-cell stage C57BL/6 J zygotes. After incubation at 37°, one-cell stage embryos were transferred into pseudopregnant ICR female mice.

### PCR screening

PCR screenings of knock-in mice were performed with primers listed in Additional file 1: Table S4 as previously described [8]. PCR products were directly sequenced or cloned, then sequenced. For flox*Col12a1* screening, PCR products were digested with HindIII (NEB).

### in vitro Cre-recombination

200 ng of PCR products of cloned flox*Col12a1* alleles from floxed mice or genomic *Col12a1* PCR products of wildtype mice were incubated with Cre recombinase (NEB) and analyzed by 2% agarose gel electrophoresis according to the manufacture’s instruction.

### Off-target effects of knock-in mice

The potential off-target candidate loci were analyzed as previously described [8]. PCR primers were listed in Additional file 1: Table S4.

### Statistical analyses

All data are presented as the mean ± SEM. Statistical methods were described in the figure legends for each data set. Statistical significance was set at *p* < 0.05.

## Additional file

Additional file 1: The following additional data are available with the online version of this paper. Additional data file 1 contains the figures including IDA analysis of PITCh-donor, PCR screenings of mice, sequencing of non-knock-in *Actb* alleles, FACS and LSM analysis of human cells, Exo1 western blotting, *Exo1* toxicity analysis, off-target analysis in mice, germline transmission, linear PCR donor injection, and sequence alignments of *hACTB* and its off-target sites, and tables of these results and a list of the oligo DNAs and RNAs used in this study. (DOCX 14447 kb)

## Abbreviations

CRISPR/Cas: Clustered regularly interspaced short palindromic repeat (CRISPR)/CRISPR-associated protein (Cas)
crRNA: Crispr RNA
DREADD: Designer receptors exclusively activated by designer drug
DSBs: Double-strand breaks
EGFP: Enhanced green fluorescent protein
*Exo1*: *Exonuclease 1*
FACS: Fluorescence-activated cell sorting
*FBL*: *Fibrillarin*
HR: Homologous recombination
IDA: In vitro digestion assay
MMEJ: Microhomology-mediated end joining
NHEJ: Nonhomologous end joining
pA: PolyA
PAM: Protospacer adjacent motif
PITCh: Precise integration into the target chromosome
sgRNA: Single-guide RNA
tetO: TetO operator
tracrRNA: Trans-activating crRNA
WPRE: Woodchuck hepatitis virus posttranscriptional regulatory element
