# Supplementary material for: Gene cassette knock-in in mammalian cells and zygotes by enhanced MMEJ
Source: BMC Genomics. 2016 Nov 28;17:979. doi: 10.1186/s12864-016-3331-9 (PMC5126809; doi:10.1186/s12864-016-3331-9)
Supplement: Additional file 1: — The following additional data are available with the online version of this paper. Additional data file 1 contains the figures including IDA analysis of PITCh-donor, PCR screenings of mice, sequencing of non-knock-in Actb alleles, FACS and LSM analysis of human cells, Exo1 western blotting, Exo1 toxicity analysis, off-target analysis in mice, germline transmission, linear PCR donor injection, and sequence alignments of hACTB and its off-target sites, and tables of these results and a list of the oligo DNAs and RNAs used in this study. (DOCX 14447 kb) [file 12864_2016_3331_MOESM1_ESM.docx]

**Additional file 1**

**Gene cassette knock-in in mammalian cells and zygotes by enhanced MMEJ**

Tomomi Aida^1,2,7,*,§^, Shota Nakade^5,*^, Tetsushi Sakuma^5,*,§^, Yayoi Izu^3^, Ayu Oishi^5,8^, Keiji Mochida^5^, Harumi Ishikubo^1,2^, Takako Usami^2^, Hidenori Aizawa^1,6^, Takashi Yamamoto^5,§^ & Kohichi Tanaka^1,4,§^

**
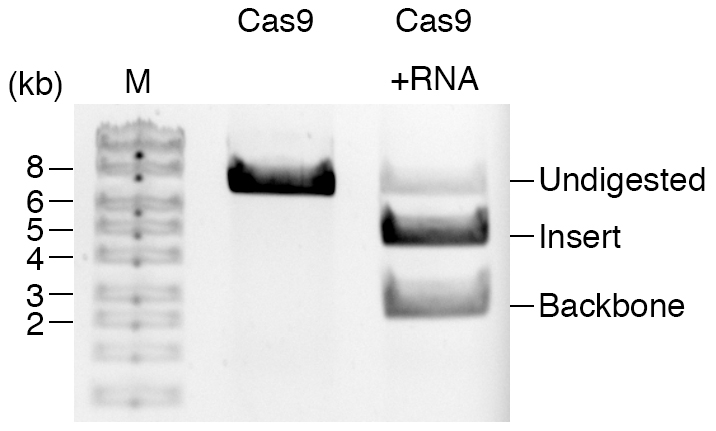
**

**Figure S1** *in vitro* digestion assay of PITCh-donor. The linearized PITCh-donor plasmids were digested by Cas9 protein with or without *gRNA-s1* crRNA and tracrRNA. M: molecular marker.

**
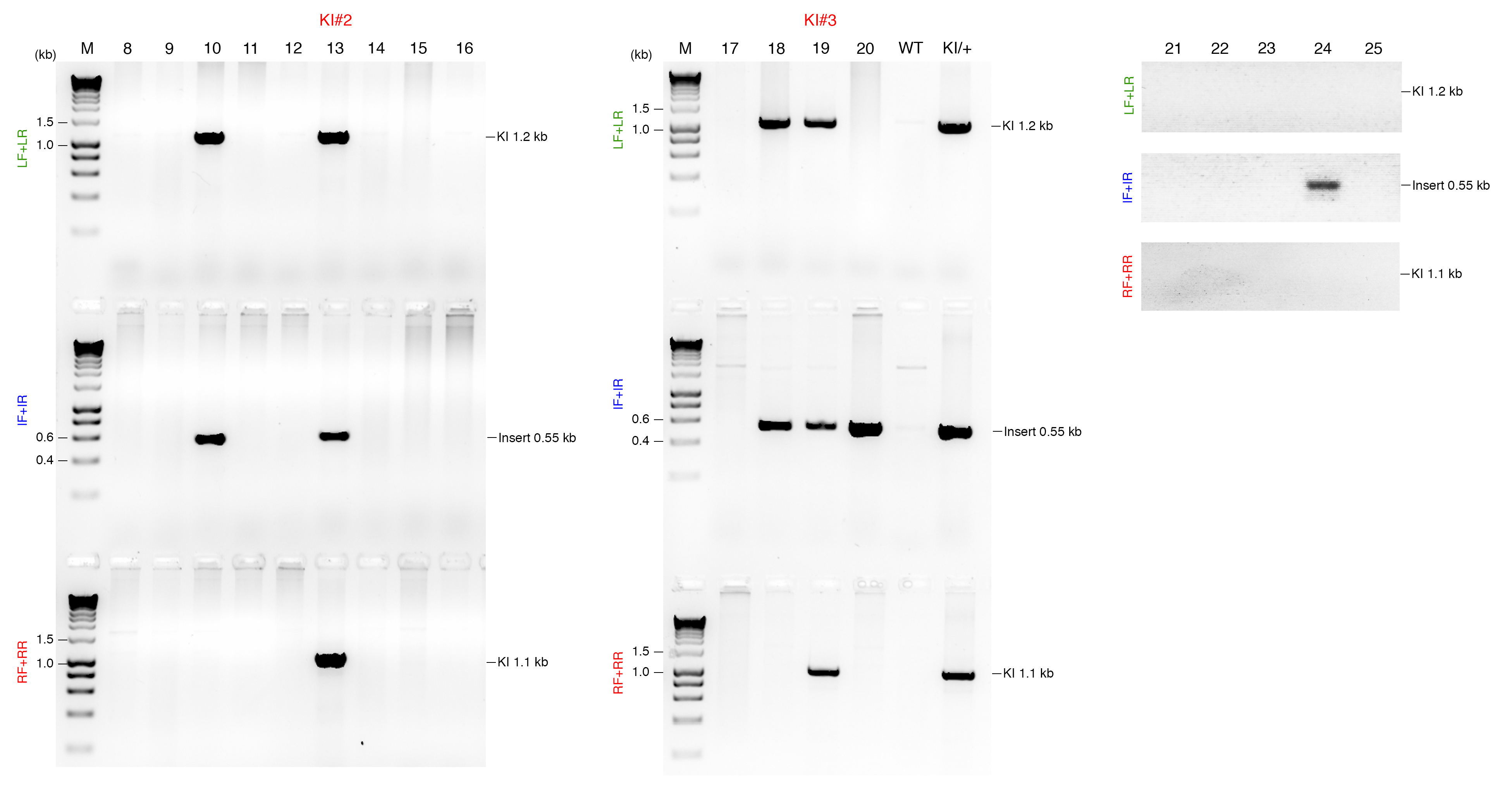
**

**Figure S2** PCR screenings of *Actb*-TetO-FLEX-hM3Dq/mCherry knock-in newborns by the PITCh system. IF: internal forward primer, IR: internal reverse primer, LF: left forward primer, LR: left reverse primer, RF: right forward primer, RR: right reverse primer, M: molecular marker, WT: wildtype, KI: knock-in, and KI/+: tail genomic DNA of F1 heterozygous knock-in pup derived from #13 (KI#2) F0 knock-in mouse.

**
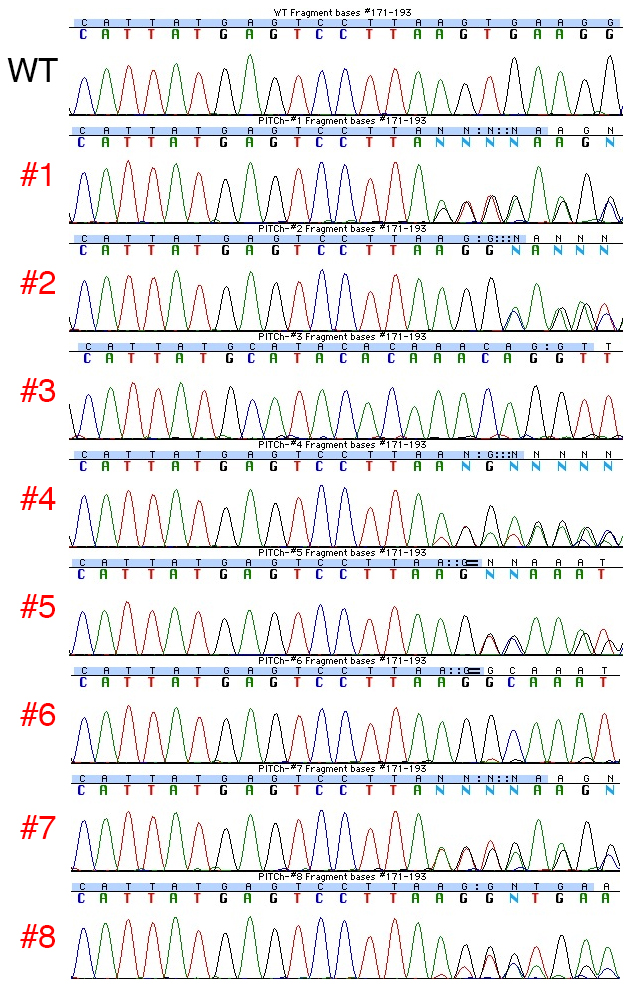
**

**Figure S3** Sequence analysis of the *Actb* non-knock-in alleles in newborn mice generated by the PITCh system corresponds to the left gel image in Figure S2 and a wildtype control (WT) corresponds to the left gel image in Figure S2. Sequences of genomic PCR shortly amplified with primers LF and RR were shown. The 20 bp target sequences and PAM are shown. Red: Modified mice.


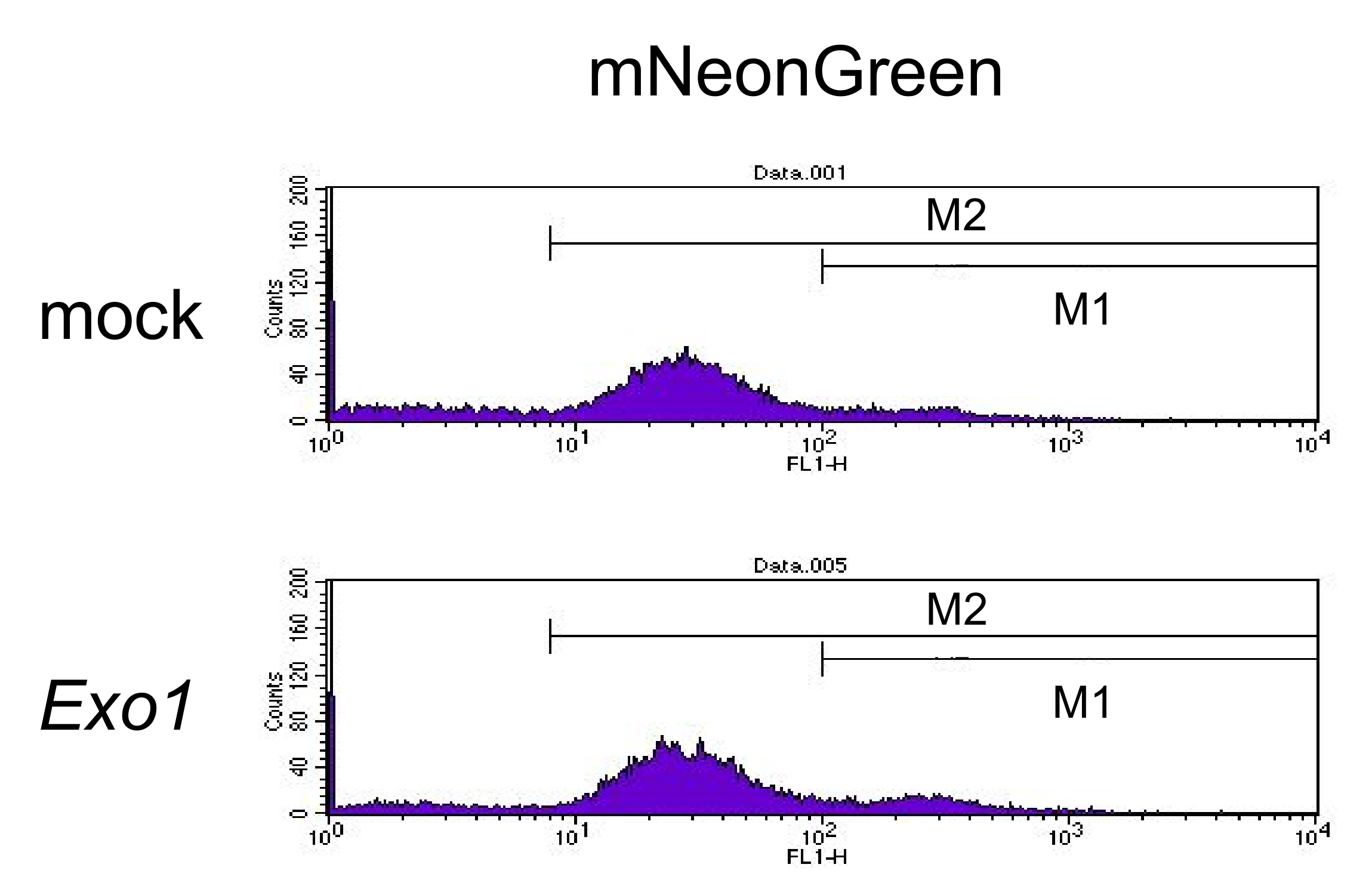


**Figure S4** Histogram images of FACS analysis, related to **Figure 3c**. All the histograms plot a single parameter (fluorescence intensity, horizontal axis) against the number of cells detected (vertical axis). The frequencies of MMEJ-mediated knock-in were calculated as the ratio of cell numbers in M1 to those in M2.


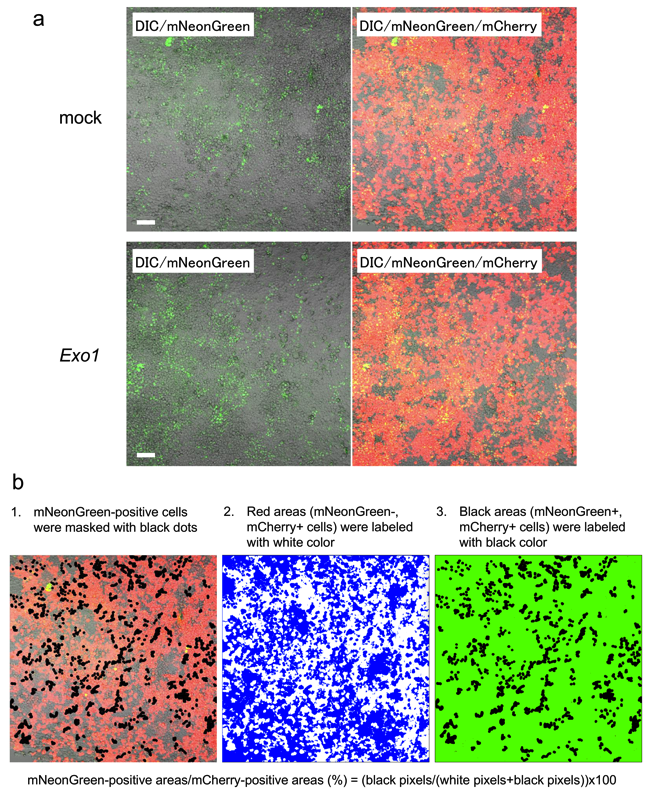


**Figure S5** Imaging analysis using LSM, related to **Figure 3d**. **(a)** LSM images of mock- and *Exo1*-overexpressed cells (upper and lower panels, respectively). Bars, 100 µm. **(b)** Summary of LSM imaging analysis. The black and white pixels were calculated using an ImageJ software.


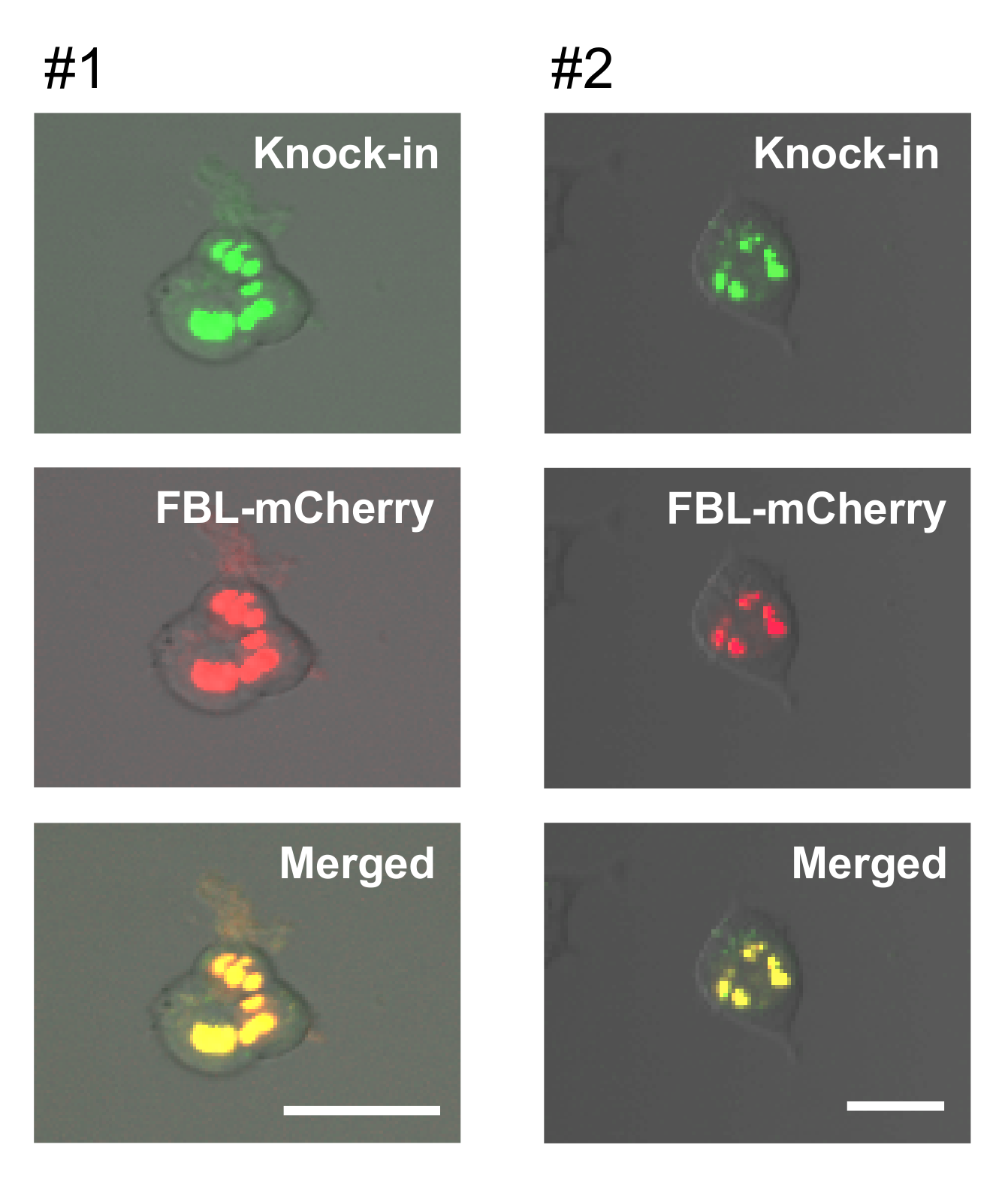


**Figure S6** LSM images of mNeonGreen knock-in cells co-transfected with *FBL-mCherry* fusion gene-expressing vectors. The fluorescence images of two independent colonies (#1 and #2) are shown. Bars, 30 µm.


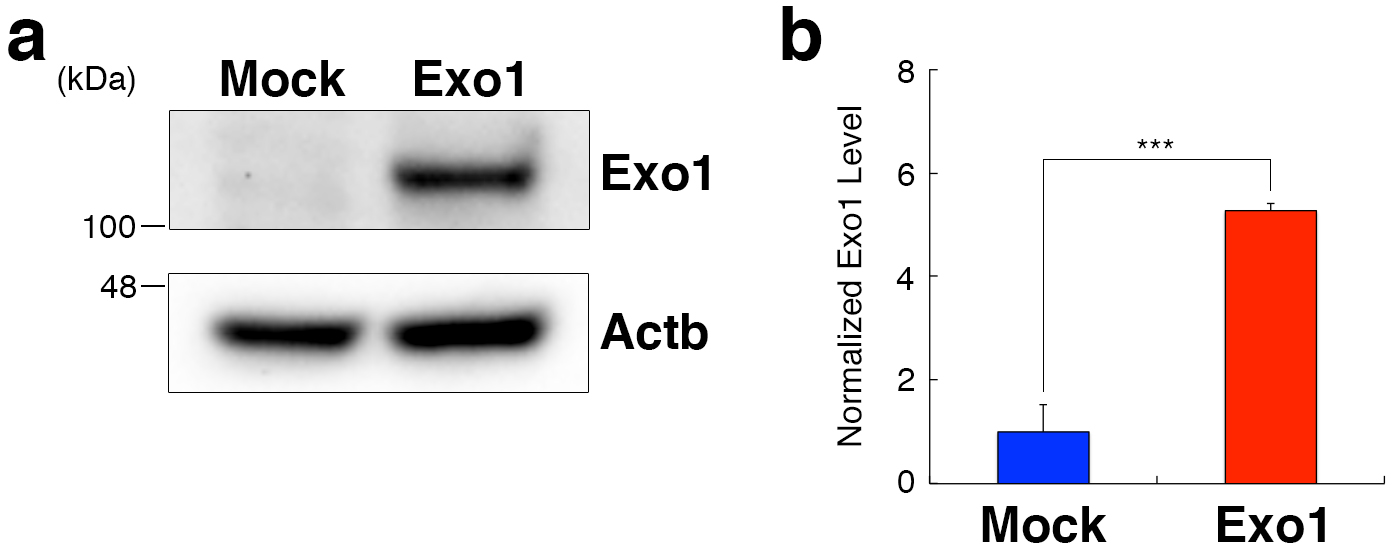


**Figure S7** Analysis of Exo1 protein expression. (**a**) Western blotting analysis of Exo1 and Actb using mock-transfected and *Exo1*-overexpressed cell lysates. (**b**) Quantification of relative protein levels of Exo1 normalized with those of Actb. Fold activation to the mock-transfected control is shown. Data are expressed as means ± SEM (n = 3). ***P < 0.005.


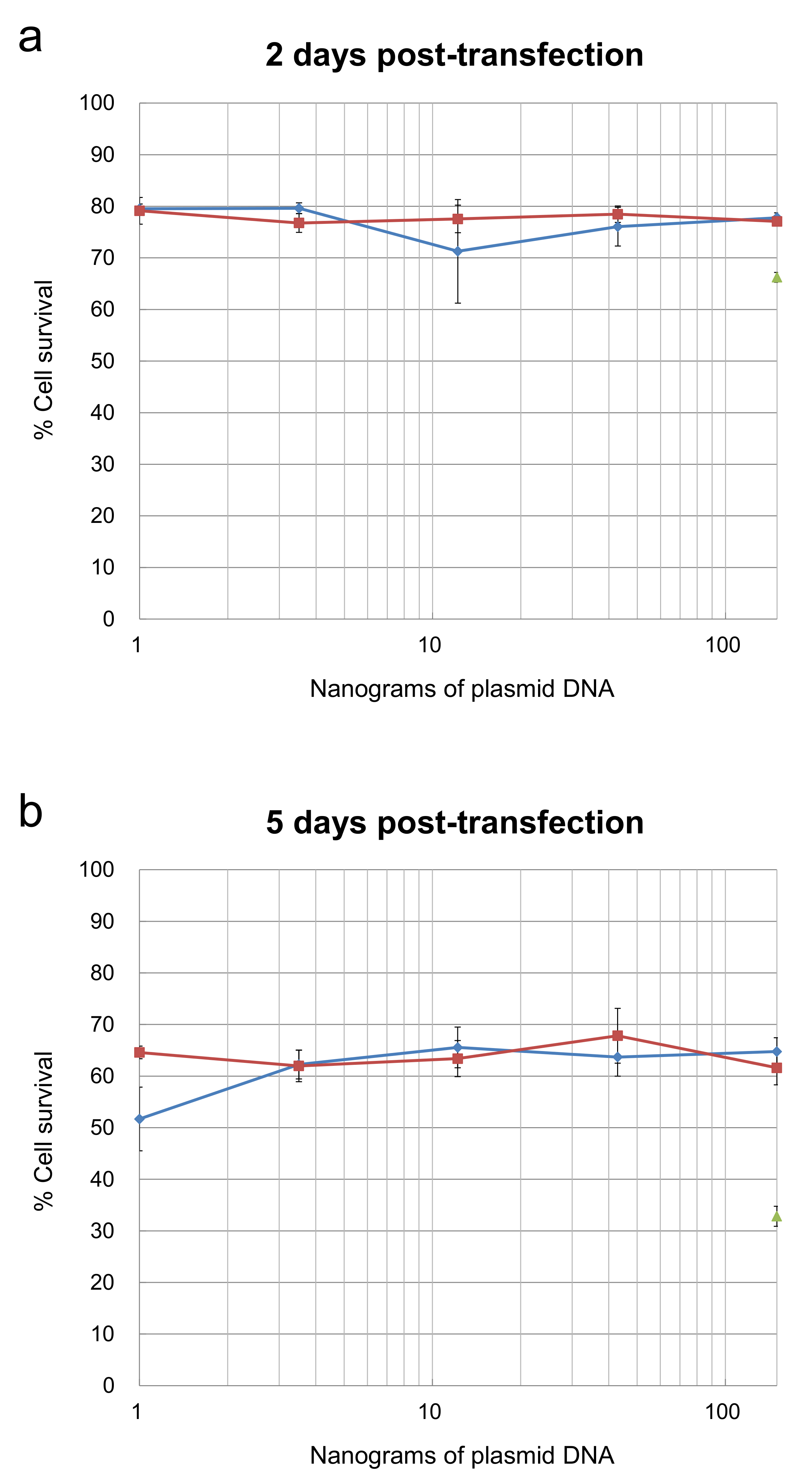


**Figure S8** Toxicity analysis of *Exo1* overexpression. The percentages of cell survival were determined at 2 (**a**) and 5 (**b**) days post-transfection. The data of mock vector-, *Exo1* vector-, and ZFN vector-transfected samples are shown in blue, red, and green, respectively. ZFN was used to validate whether the cell toxicity can be measured in our experiment, because it has been reported to be toxic in human cells [49]. Data are expressed as means ± SEM (n = 3).

**
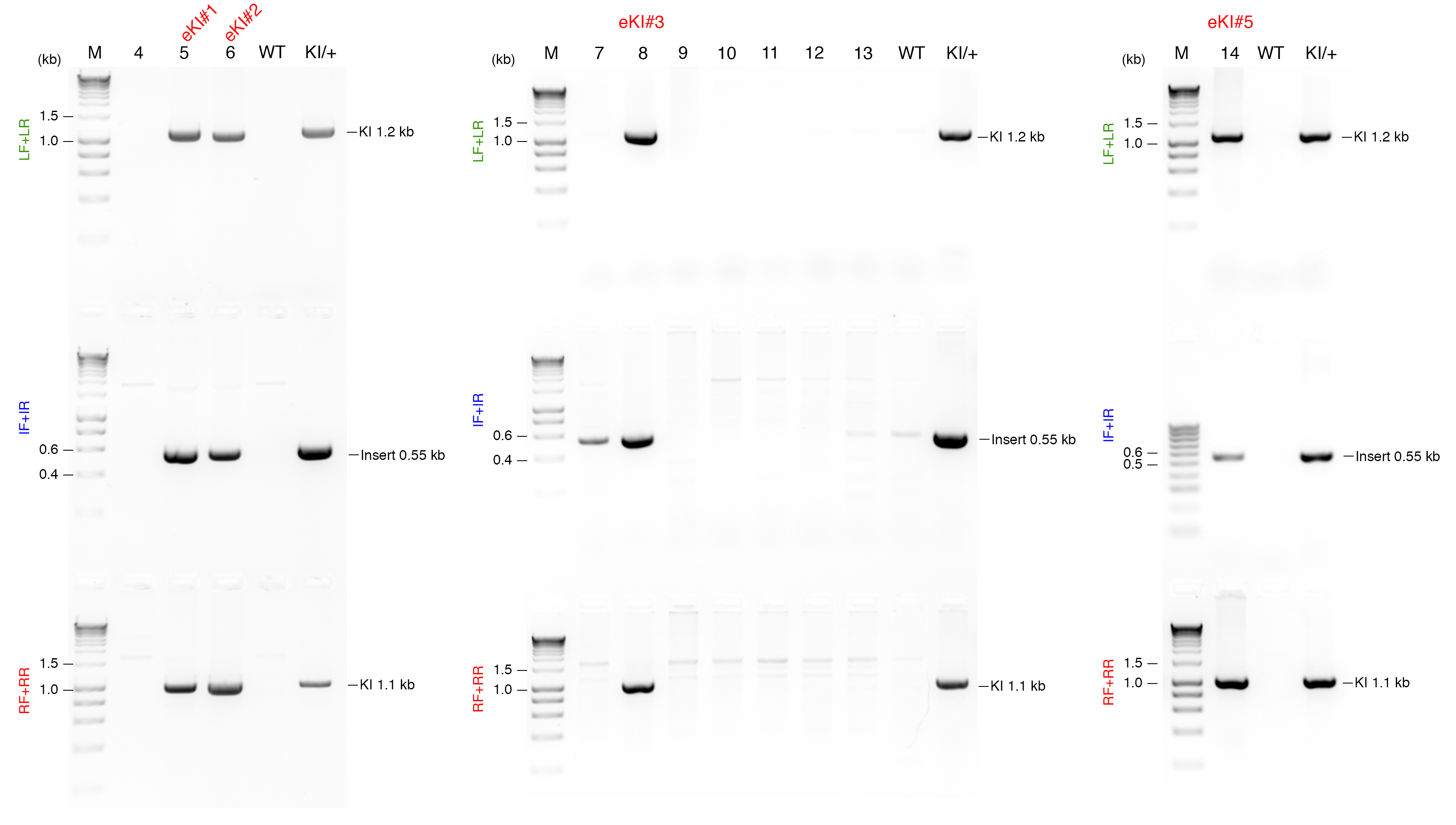
**

**Figure S9** PCR screenings of *Actb*-TetO-FLEX-hM3Dq/mCherry knock-in newborns by the enhanced PITCh system. IF: internal forward primer, IR: internal reverse primer, LF: left forward primer, LR: left reverse primer, RF: right forward primer, RR: right reverse primer, M: molecular marker, WT: wildtype, KI: knock-in, and KI/+: tail genomic DNA of F1 heterozygous knock-in pup derived from #13 (KI#2) F0 knock-in mouse.
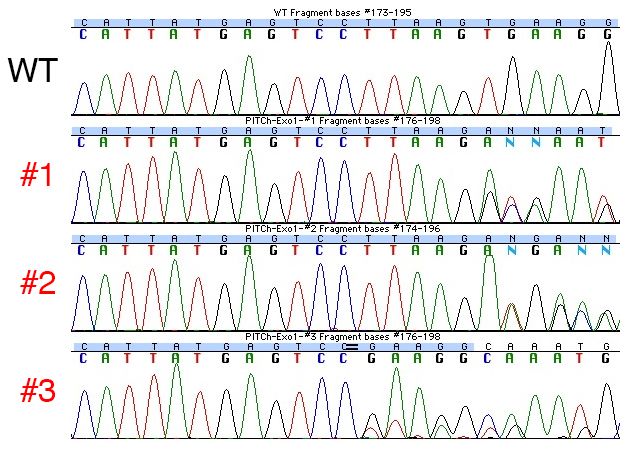


**Figure S10** Sequence analysis of the *Actb* non-knock-in alleles in newborn mice generated by the enhanced PITCh system and a wildtype control (WT) corresponds to the left gel image in Figure S9. Sequences of genomic PCR shortly amplified with primers LF and RR were shown. The 20 bp target sequences and PAM are shown. Red: Modified mice.


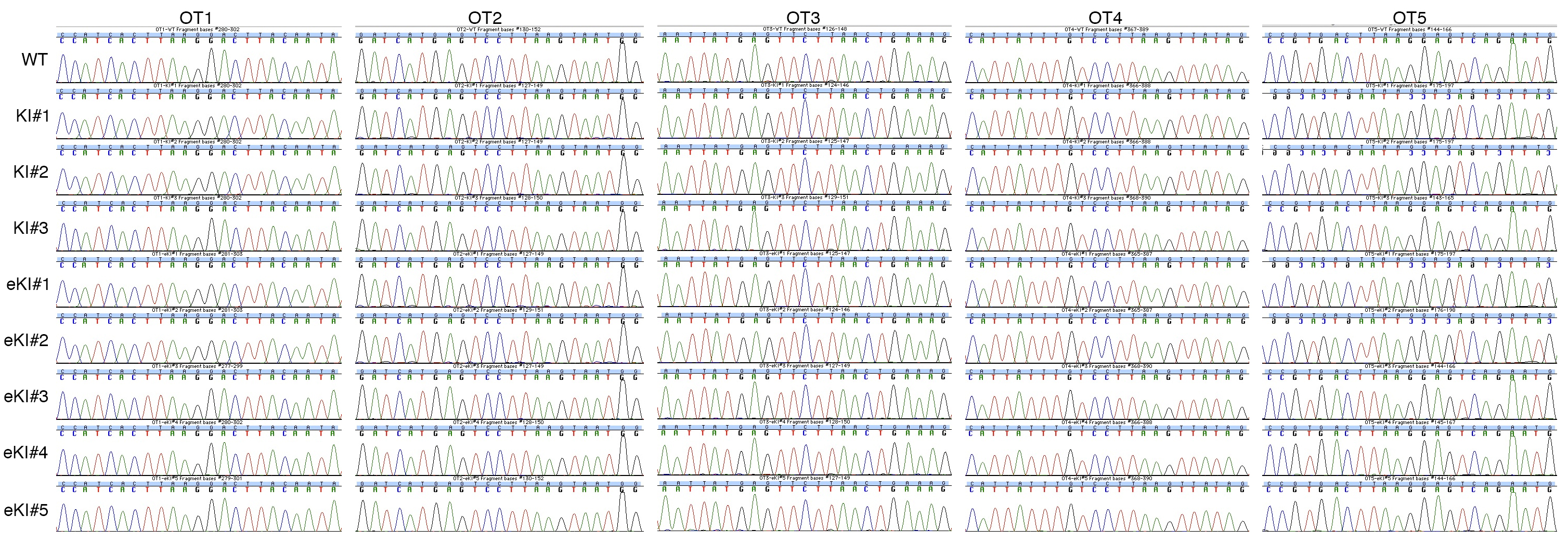


**Figure S11** Sequencing of 5 off-target candidate loci in 8 knock-in newborns and a wildtype control. The 20 bp target sequences and PAM are shown.


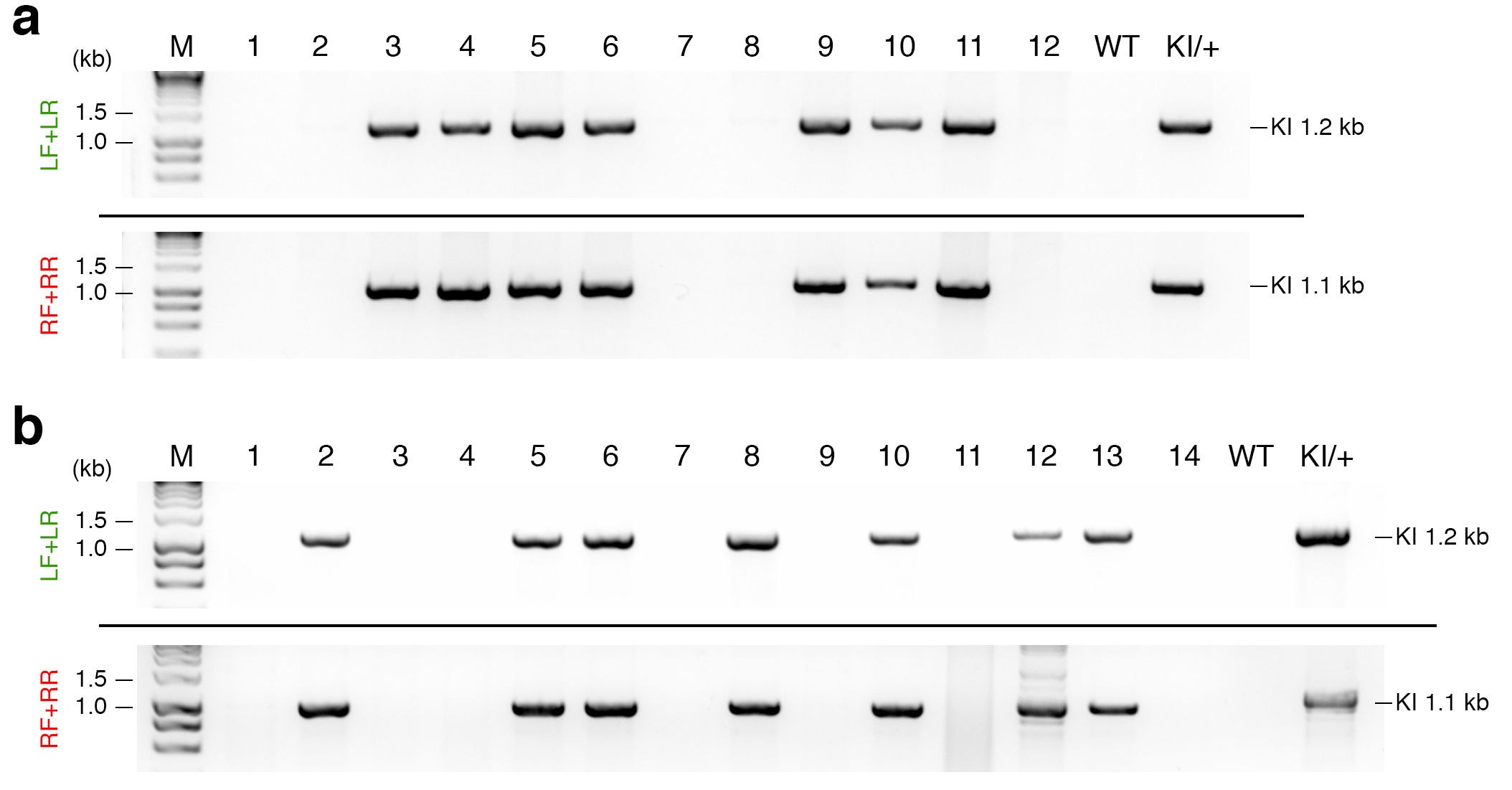


**Figure S12** Analysis of germline transmission of the knock-in allele by PCR. Gel images of PCR genotyping of F1 progenies derived from **(a)** KI#1 and **(b)** eKI#5 are shown. LF: left forward primer, LR: left reverse primer, RF: right forward primer, RR: right reverse primer, WT: wildtype, KI: knock-in, M: molecular marker, and KI/+: tail genomic DNA of F1 heterozygous knock-in pup derived from #13 (KI#2) F0 knock-in mouse.


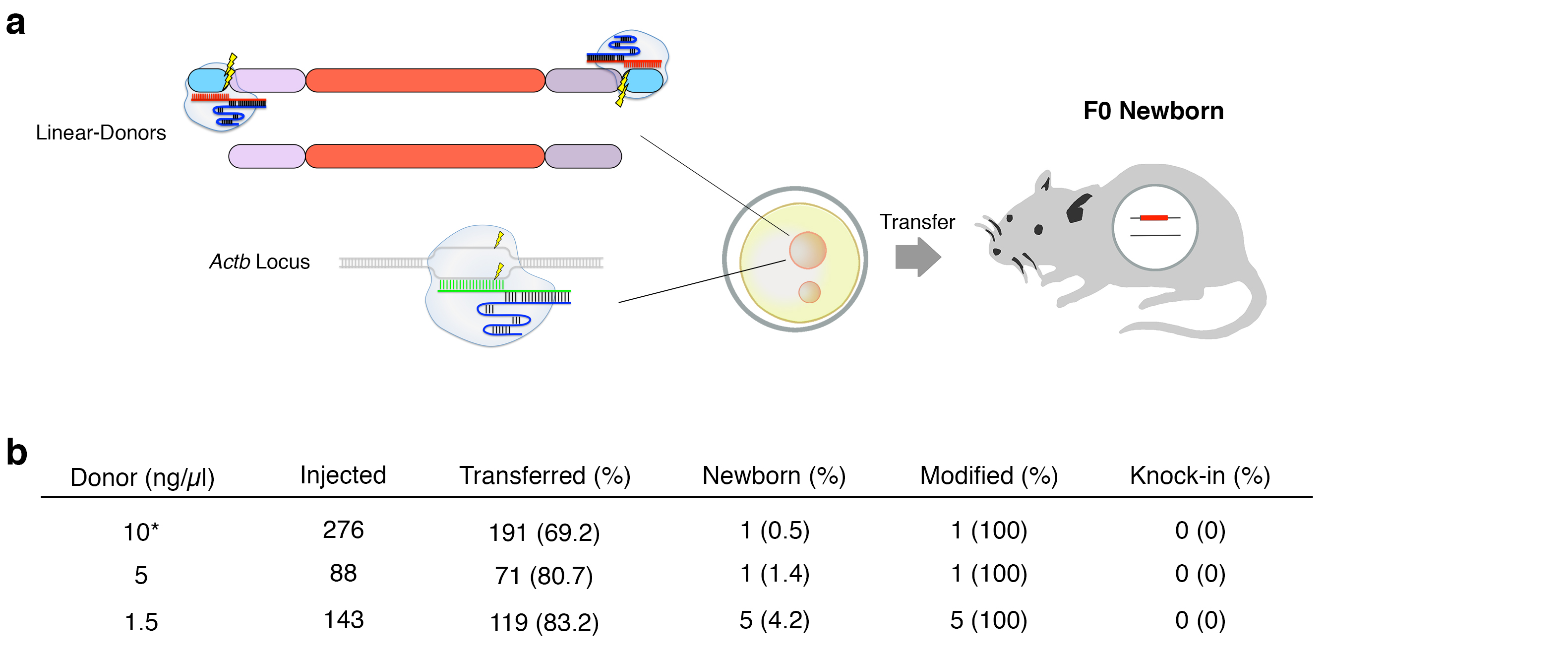


**Figure S13 (a)** Schematic diagram of pronuclear injection of Cas9 protein, *Actb* crRNA (and *gRNA-s1* crRNA), tracrRNA and linear PCR-PITCh-donor. The red, purple, and blue boxes indicate the insert, *Actb* microhomologies, and *gRNA-s1* target sequences, respectively. **(b)** Summary of *Actb*-TetO-FLEX-hM3Dq/mCherry knock-in mouse production by linear PCR-PITCh-donor injections. *Linear PCR-PITCh-donor containing *gRNA-s1* target sites (upper linear-donor in **a**) was used with *gRNA-s1* crRNA for *in situ* digestion of the donor.


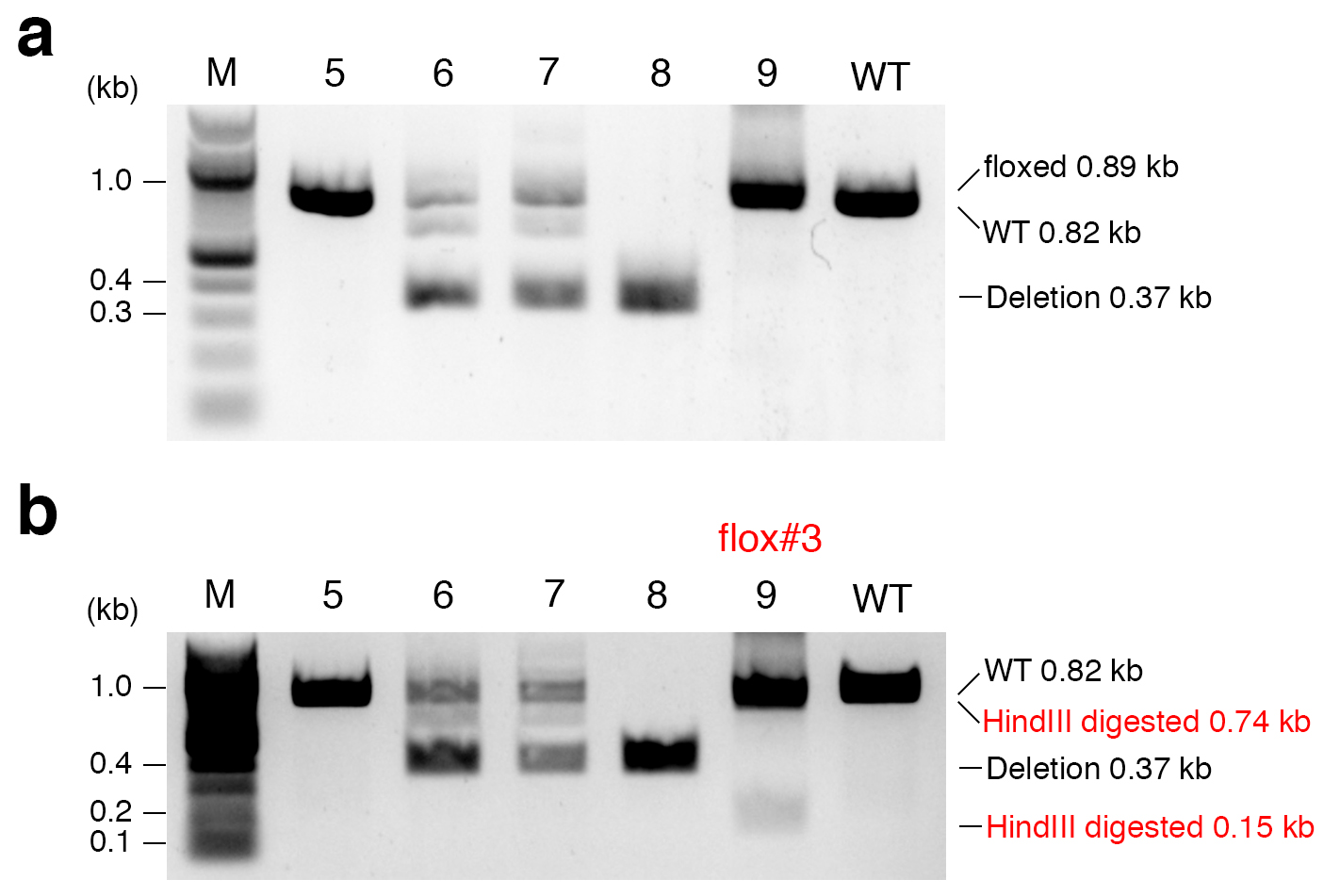


**Figure S14 (a)** PCR screenings of flox*Col12a1* newborns. **(b)** PCR-RFLP screenings of flox*Col12a1* newborn mice. M: molecular marker, and WT: wildtype.


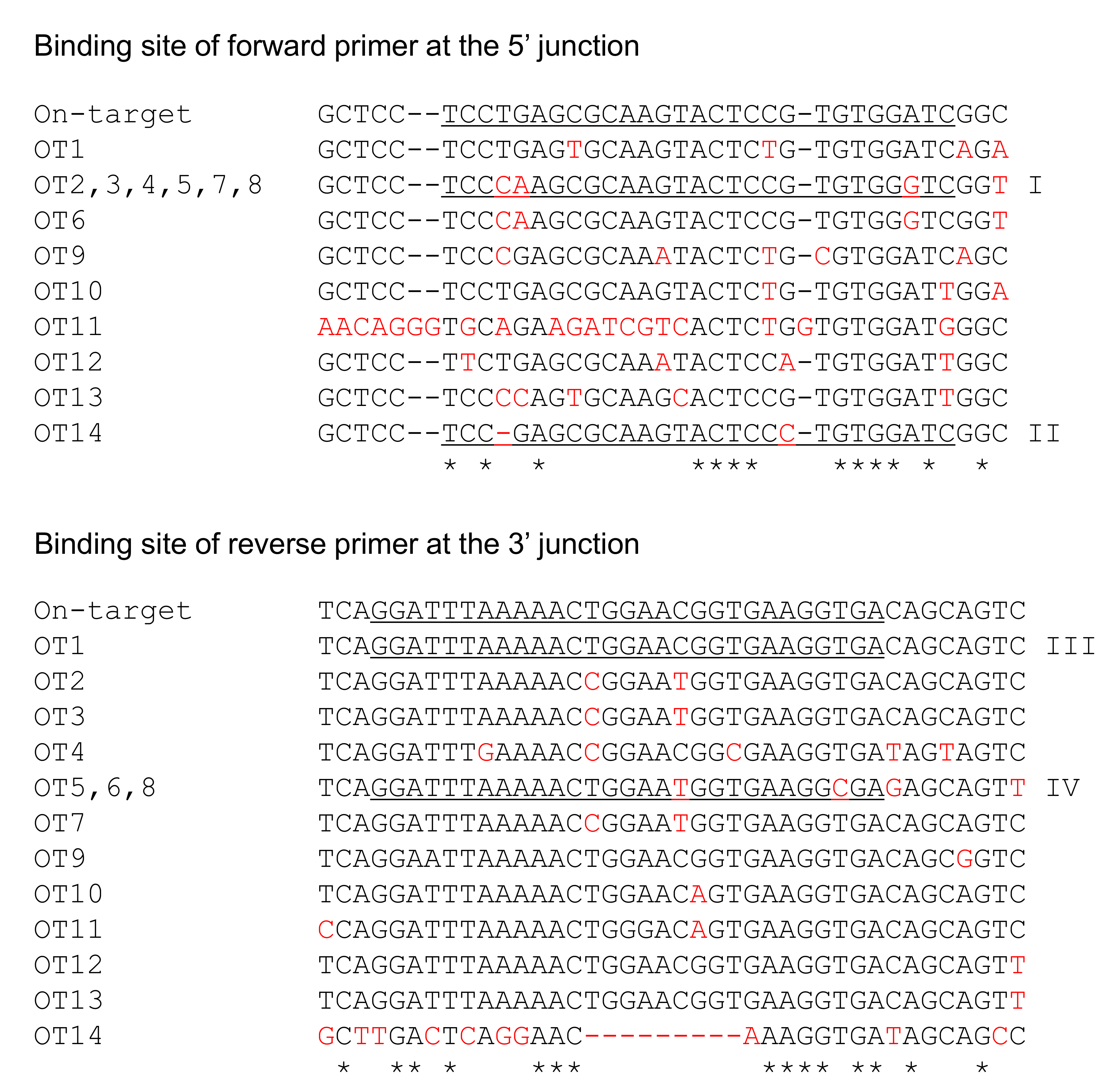


**Figure S15** Sequence alignment of primer-binding sites of 5’ and 3’ junctions, related to **Figure 4d, 4e, 5a,** **S16** and **Table S1**. Red letters indicate mismatched bases. Roman numbers shown at the right side of each set of sequences indicate the allele types. Asterisks indicate positions with complete base conservation among on- and off-target sites. The primer-binding sequences were underlined.


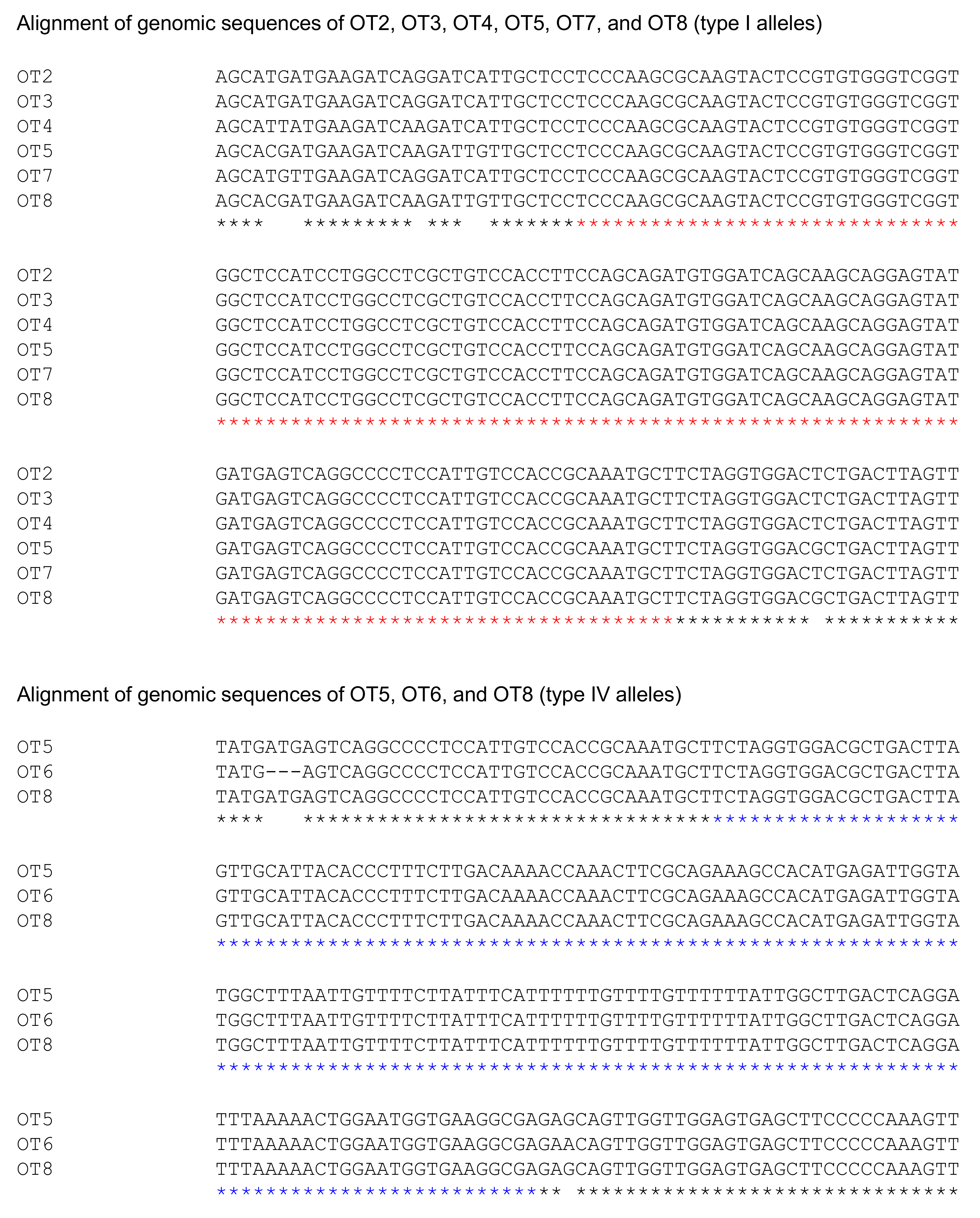


**Figure S16** Sequence alignment of type I alleles (OT2, OT3, OT4, OT5, OT7, and OT8) and type IV alleles (OT5, OT6, and OT8), related to **Figure 4d, 4e, 5a,** **S15** and **Table S1**. Asterisks indicate positions with complete base conservation among on- and off-target sites. Red letters indicate the region from the binding site of forward primer to the DSB site. Blue letters indicate the region from the DSB site to the binding site of reverse primer. The sequence identity of type I and IV alleles within these regions was 100%.

>MMEJ reporter vector (Red: CMV enhancer/promoter, Green: mutated EGFP)

AACAACACTCAACCCTATCTCGGTCTATTCTTTTGATTTATAAGGGATTTTGGGGATTTCGGCCTATTGGTTAAAAAATGAGCTGATTTAACAAAAATTTAACGCGAATTAATTCTGTGGAATGTGTGTCAGTTAGGGTGTGGAAAGTCCCCAGGCTCCCCAGGCAGGCAGAAGTATGCAAAGCATGCATCTCAATTAGTCAGCAACCAGGTGTGGAAAGTCCCCAGGCTCCCCAGCAGGCAGAAGTATGCAAAGCATGCATCTCAATTAGTCAGCAACCATAGTCCCGCCCCTAACTCCGCCCATCCCGCCCCTAACTCCGCCCAGTTCCGCCCATTCTCCGCCCCATGGCTGACTAATTTTTTTTATTTATGCAGAGGCCGAGGCCGCCTCTGCCTCTGAGCTATTCCAGAAGTAGTGAGGAGGCTTTTTTGGAGGCCTAGGCTTTTGCAAAAAGCTCCCGGGAGCTTGTATATCCATTTTCGGATCTGATCAAGAGACAGGATGAGGATCGTTTCGCATGATTGAACAAGATGGATTGCACGCAGGTTCTCCGGCCGCTTGGGTGGAGAGGCTATTCGGCTATGACTGGGCACAACAGACAATCGGCTGCTCTGATGCCGCCGTGTTCCGGCTGTCAGCGCAGGGGCGCCCGGTTCTTTTTGTCAAGACCGACCTGTCCGGTGCCCTGAATGAACTGCAGGACGAGGCAGCGCGGCTATCGTGGCTGGCCACGACGGGCGTTCCTTGCGCAGCTGTGCTCGACGTTGTCACTGAAGCGGGAAGGGACTGGCTGCTATTGGGCGAAGTGCCGGGGCAGGATCTCCTGTCATCTCACCTTGCTCCTGCCGAGAAAGTATCCATCATGGCTGATGCAATGCGGCGGCTGCATACGCTTGATCCGGCTACCTGCCCATTCGACCACCAAGCGAAACATCGCATCGAGCGAGCACGTACTCGGATGGAAGCCGGTCTTGTCGATCAGGATGATCTGGACGAAGAGCATCAGGGGCTCGCGCCAGCCGAACTGTTCGCCAGGCTCAAGGCGCGCATGCCCGACGGCGAGGATCTCGTCGTGACCCATGGCGATGCCTGCTTGCCGAATATCATGGTGGAAAATGGCCGCTTTTCTGGATTCATCGACTGTGGCCGGCTGGGTGTGGCGGACCGCTATCAGGACATAGCGTTGGCTACCCGTGATATTGCTGAAGAGCTTGGCGGCGAATGGGCTGACCGCTTCCTCGTGCTTTACGGTATCGCCGCTCCCGATTCGCAGCGCATCGCCTTCTATCGCCTTCTTGACGAGTTCTTCTGAGCGGGACTCTGGGGTTCGCGAAATGACCGACCAAGCGACGCCCAACCTGCCATCACGAGATTTCGATTCCACCGCCGCCTTCTATGAAAGGTTGGGCTTCGGAATCGTTTTCCGGGACGCCGGCTGGATGATCCTCCAGCGCGGGGATCTCATGCTGGAGTTCTTCGCCCACCCCAACTTGTTTATTGCAGCTTATAATGGTTACAAATAAAGCAATAGCATCACAAATTTCACAAATAAAGCATTTTTTTCACTGCATTCTAGTTGTGGTTTGTCCAAACTCATCAATGTATCTTATCATGTCTGTATACCGTCGACCTCTAGCTAGAGCTTGGCGTAATCATGGTCATAGCTGTTTCCTGTGTGAAATTGTTATCCGCTCACAATTCCACACAACATACGAGCCGGAAGCATAAAGTGTAAAGCCTGGGGTGCCTAATGAGTGAGCTAACTCACATTAATTGCGTTGCGCTCACTGCCCGCTTTCCAGTCGGGAAACCTGTCGTGCCAGCTGCATTAATGAATCGGCCAACGCGCGGGGAGAGGCGGTTTGCGTATTGGGCGCTCTTCCGCTTCCTCGCTCACTGACTCGCTGCGCTCGGTCGTTCGGCTGCGGCGAGCGGTATCAGCTCACTCAAAGGCGGTAATACGGTTATCCACAGAATCAGGGGATAACGCAGGAAAGAACATGTGAGCAAAAGGCCAGCAAAAGGCCAGGAACCGTAAAAAGGCCGCGTTGCTGGCGTTTTTCCATAGGCTCCGCCCCCCTGACGAGCATCACAAAAATCGACGCTCAAGTCAGAGGTGGCGAAACCCGACAGGACTATAAAGATACCAGGCGTTTCCCCCTGGAAGCTCCCTCGTGCGCTCTCCTGTTCCGACCCTGCCGCTTACCGGATACCTGTCCGCCTTTCTCCCTTCGGGAAGCGTGGCGCTTTCTCAATGCTCACGCTGTAGGTATCTCAGTTCGGTGTAGGTCGTTCGCTCCAAGCTGGGCTGTGTGCACGAACCCCCCGTTCAGCCCGACCGCTGCGCCTTATCCGGTAACTATCGTCTTGAGTCCAACCCGGTAAGACACGACTTATCGCCACTGGCAGCAGCCACTGGTAACAGGATTAGCAGAGCGAGGTATGTAGGCGGTGCTACAGAGTTCTTGAAGTGGTGGCCTAACTACGGCTACACTAGAAGGACAGTATTTGGTATCTGCGCTCTGCTGAAGCCAGTTACCTTCGGAAAAAGAGTTGGTAGCTCTTGATCCGGCAAACAAACCACCGCTGGTAGCGGTGGTTTTTTTGTTTGCAAGCAGCAGATTACGCGCAGAAAAAAAGGATCTCAAGAAGATCCTTTGATCTTTTCTACGGGGTCTGACGCTCAGTGGAACGAAAACTCACGTTAAGGGATTTTGGTCATGAGATTATCAAAAAGGATCTTCACCTAGATCCTTTTAAATTAAAAATGAAGTTTTAAATCAATCTAAAGTATATATGAGTAAACTTGGTCTGACAGTTACCAATGCTTAATCAGTGAGGCACCTATCTCAGCGATCTGTCTATTTCGTTCATCCATAGTTGCCTGACTCCCCGTCGTGTAGATAACTACGATACGGGAGGGCTTACCATCTGGCCCCAGTGCTGCAATGATACCGCGAGACCCACGCTCACCGGCTCCAGATTTATCAGCAATAAACCAGCCAGCCGGAAGGGCCGAGCGCAGAAGTGGTCCTGCAACTTTATCCGCCTCCATCCAGTCTATTAATTGTTGCCGGGAAGCTAGAGTAAGTAGTTCGCCAGTTAATAGTTTGCGCAACGTTGTTGCCATTGCTACAGGCATCGTGGTGTCACGCTCGTCGTTTGGTATGGCTTCATTCAGCTCCGGTTCCCAACGATCAAGGCGAGTTACATGATCCCCCATGTTGTGCAAAAAAGCGGTTAGCTCCTTCGGTCCTCCGATCGTTGTCAGAAGTAAGTTGGCCGCAGTGTTATCACTCATGGTTATGGCAGCACTGCATAATTCTCTTACTGTCATGCCATCCGTAAGATGCTTTTCTGTGACTGGTGAGTACTCAACCAAGTCATTCTGAGAATAGTGTATGCGGCGACCGAGTTGCTCTTGCCCGGCGTCAATACGGGATAATACCGCGCCACATAGCAGAACTTTAAAAGTGCTCATCATTGGAAAACGTTCTTCGGGGCGAAAACTCTCAAGGATCTTACCGCTGTTGAGATCCAGTTCGATGTAACCCACTCGTGCACCCAACTGATCTTCAGCATCTTTTACTTTCACCAGCGTTTCTGGGTGAGCAAAAACAGGAAGGCAAAATGCCGCAAAAAAGGGAATAAGGGCGACACGGAAATGTTGAATACTCATACTCTTCCTTTTTCAATATTATTGAAGCATTTATCAGGGTTATTGTCTCATGAGCGGATACATATTTGAATGTATTTAGAAAAATAAACAAATAGGGGTTCCGCGCACATTTCCCCGAAAAGTGCCACCTGACGTCGACGGATCGGGAGATCTCCCGATCCCCTATGGTCGACTCTCAGTACAATCTGCTCTGATGCCGCATAGTTAAGCCAGTATCTGCTCCCTGCTTGTGTGTTGGAGGTCGCTGAGTAGTGCGCGAGCAAAATTTAAGCTACAACAAGGCAAGGCTTGACCGACAATTGCATGAAGAATCTGCTTAGGGTTAGGCGTTTTGCGCTGCTTCGCGATGTACGGGCCAGATATACGCGTTGACATTGATTATTGACTAGTTATTAATAGTAATCAATTACGGGGTCATTAGTTCATAGCCCATATATGGAGTTCCGCGTTACATAACTTACGGTAAATGGCCCGCCTGGCTGACCGCCCAACGACCCCCGCCCATTGACGTCAATAATGACGTATGTTCCCATAGTAACGCCAATAGGGACTTTCCATTGACGTCAATGGGTGGACTATTTACGGTAAACTGCCCACTTGGCAGTACATCAAGTGTATCATATGCCAAGTACGCCCCCTATTGACGTCAATGACGGTAAATGGCCCGCCTGGCATTATGCCCAGTACATGACCTTATGGGACTTTCCTACTTGGCAGTACATCTACGTATTAGTCATCGCTATTACCATGGTGATGCGGTTTTGGCAGTACATCAATGGGCGTGGATAGCGGTTTGACTCACGGGGATTTCCAAGTCTCCACCCCATTGACGTCAATGGGAGTTTGTTTTGGCACCAAAATCAACGGGACTTTCCAAAATGTCGTAACAACTCCGCCCCATTGACGCAAATGGGCGGTAGGCGTGTACGGTGGGAGGTCTATATAAGCAGAGCTCTCTGGCTAACTAGAGAACCCACTGCTTACTGGCTTATCGAAATTAATACGACTCACTATAGGGAGACCCAAGCTGGCTAGTTAAGCTTGCTTGTTCTTTTTGCAGAAGCTCAGAATAAACGCTCAACTTTGGCAGATAAGCTCGGATCTAACTCGAGAAAGATATTGTATATATCGTAACAATAGGAGGTTCAACAATGGTGAGCAAGGGCGAGGAGCTGTTCACCGGGGTGGTGCCCATCCTGGTCGAGCTGGACGGCGACGTAAACGGCCACAAGTTCAGCGTGTCCGGCGAGGGCGAGGGCGATGCCACCTACGGCAAGCTGACCCTGAAGTTCATCTGCACCACCGGCAAGCTGCCCGTGGCCCACCCTCGTGACCACACTGACCTACGACTGACCTACGGCGTGCAGTGCTTCAGCCGCTACCCCGACCACATGAAGCAGCACGACTTCTTCAAGTCCGCCATGCCCGAAGGCTACGTCCAGGAGCGCACCATCTTCTTCAAGGACGACGGCAACTACAAGACCCGCGCCGAGGTGAAGTTCGAGGGCGACACCCTGGTGAACCGCATCGAGCTGAAGGGCATCGACTTCAAGGAGGACGGCAACATCCTGGGGCACAAGCTGGAGTACAACTACAACAGCCACAACGTCTATATCATGGCCGACAAGCAGAAGAACGGCATCAAGGTGAACTTCAAGATCCGCCACAACATCGAGGACGGCAGCGTGCAGCTCGCCGACCACTACCAGCAGAACACCCCCATCGGCGACGGCCCCGTGCTGCTGCCCGACAACCACTACCTGAGCACCCAGTCCGCCCTGAGCAAAGACCCCAACGAGAAGCGCGATCACATGGTCCTGCTGGAGTTCGTGACCGCCGCCGGGATCACTCTCGGCATGGACGAGCTGTACAAGTAATAGGAAACCCGCTGATCAGCCTCGACTGTGCCTTCTAGTTGCCAGCCATCTGTTGTTTGCCCCTCCCCCGTGCCTTCCTTGACCCTGGAAGGTGCCACTCCCACTGTCCTTTCCTAATAAAATGAGGAAATTGCATCGCATTGTCTGAGTAGGTGTCATTCTATTCTGGGGGGTGGGGTGGGGCAGGACAGCAAGGGGGAGGATTGGGAAGACAATAGCAGGCATGCTGGGGATGCGGTGGGCTCTATGGCTTCTGAGGCGGAAAGAACCAGCTGGGGCTCTAGGGGGTATCCCCACGCGCCCTGTAGCGGCGCATTAAGCGCGGCGGGTGTGGTGGTTACGCGCAGCGTGACCGCTACACTTGCCAGCGCCCTAGCGCCCGCTCCTTTCGCTTTCTTCCCTTCCTTTCTCGCCACGTTCGCCGGCTTTCCCCGTCAAGCTCTAAATCGGGGCATCCCTTTAGGGTTCCGATTTAGTGCTTTACGGCACCTCGACCCCAAAAAACTTGATTAGGGTGATGGTTCACGTAGTGGGCCATCGCCCTGATAGACGGTTTTTCGCCCTTTGACGTTGGAGTCCACGTTCTTTAATAGTGGACTCTTGTTCCAAACTGG

> PITCh(*gRNA-s1*)-*FBL* (Green: mNeonGreen, Orange: 2A, Blue: Puro)

GACGAAAGGGCCTCGTGATACGCCTATTTTTATAGGTTAATGTCATGATAATAATGGTTTCTTAGACGTCAGGTGGCACTTTTCGGGGAAATGTGCGCGGAACCCCTATTTGTTTATTTTTCTAAATACATTCAAATATGTATCCGCTCATGAGACAATAACCCTGATAAATGCTTATAATAATATTGAAAAAGGAAGAGTATGAGTATTCAACATTTCCGTGTCGCCCTTATTCCCTTTTTTGCGGCATTTTGCCTTCCTGTTTTTGCTCACCCAGAAACGCTGGTGAAAGTAAAAGATGCTGAAGATCAGTTGGGTGCACGAGTGGGTTACATCGAACTGGATCTCAACAGCGGTAAGATCCTTGAGAGTTTTCGCCCCGAAGAACGTTTTCCAATGATGAGCACTTTTAAAGTTCTGCTATGTGGCGCGGTATTATCCCGTATTGACGCCGGGCAAGAGCAACTCGGTCGCCGCATACACTATTCTCAGAATGACTTGGTTGAGTACTCACCAGTCACAGAAAAGCATCTTACGGATGGCATGACAGTAAGAGAATTATGCAGTGCTGCCATAACCATGAGTGATAACACTGCGGCCAACTTACTTCTGACAACGATCGGAGGACCGAAGGAGCTAACCGCTTTTTTGCACAACATGGGGGATCATGTAACTCGCCTTGATCGTTGGGAACCGGAGCTGAATGAAGCCATACCAAACGACGAGCGTGACACCACGATGCCTGTAGCAATGGCAACAACGTTGCGCAAACTATTAACTGGCGAACTACTTACTCTAGCTTCCCGGCAACAATTAATAGACTGGATGGAGGCGGATAAAGTTGCAGGACCACTTCTGCGCTCGGCCCTTCCGGCTGGCTGGTTTATTGCTGATAAATCTGGAGCCGGTGAGCGTGGGTCTCGCGGTATCATTGCAGCACTGGGGCCAGATGGTAAGCCCTCCCGTATCGTAGTTATCTACACGACGGGGAGTCAGGCAACTATGGATGAACGAAATAGACAGATCGCTGAGATAGGTGCCTCACTGATTAAGCATTGGTAACTGTCAGACCAAGTTTAAACATATATACTTTAGATTGATTTAAAACTTCATTTTTAATTTAAAAGGATCTAGGTGAAGATCCTTTTTGATAATCTCATGACCAAAATCCCTTAACGTGAGTTTTCGTTCCACTGAGCGTCAGACCCCGTAGAAAAGATCAAAGGATCTTCTTGAGATCCTTTTTTTCTGCGCGTAATCTGCTGCTTGCAAACAAAAAAACCACCGCTACCAGCGGTGGTTTGTTTGCCGGATCAAGAGCTACCAACTCTTTTTCCGAAGGTAACTGGCTTCAGCAGAGCGCAGATACCAAATACTGTTCTTCTAGTGTAGCCGTAGTTAGGCCACCACTTCAAGAACTCTGTAGCACCGCCTACATACCTCGCTCTGCTAATCCTGTTACCAGTGGCTGCTGCCAGTGGCGATAAGTCGTGTCTTACCGGGTTGGACTCAAGACGATAGTTACCGGATAAGGCGCAGCGGTCGGGCTGAACGGGGGGTTCGTGCACACAGCCCAGCTTGGAGCGAACGACCTACACCGAACTGAGATACCTACAGCGTGAGCTATGAGAAAGCGCCACGCTTCCCGAAGGGAGAAAGGCGGACAGGTATCCGGTAAGCGGCAGGGTCGGAACAGGAGAGCGCACGAGGGAGCTTCCAGGGGGAAACGCCTGGTATCTTTATAGTCCTGTCGGGTTTCGCCACCTCTGACTTGAGCGTCGATTTTTGTGATGCTCGTCAGGGGGGCGGAGCCTATGGAAAAACGCCAGCAACGCGGCCTTTTTACGGTTCCTGGCCTTTTGCTGGCCTTTTGCTCACATGTTCTTTCCTGCGTTATCCCCTGATTCTGTGGATAACCGTATTACCGCCTTTGAGTGAGCTGATACCGCTCGCCGCAGCCGAACGACCGAGCGCAGCGAGTCAGTGAGCGAGGAAGCGGAAGAGCGCCCAATACGCAAACCGCCTCTCCCCGCGCGTTGGCCGATTCATTAATGCAGCTGGCACGACAGGTTTCCCGACTGGAAAGCGGGCAGTGAGCGCAACGCAATTAATGTGAGTTAGCTCACTCATTAGGCACCCCAGGCTTTACACTTTATGCTTCCGGCTCGTATGTTGTGTGGAATTGTGAGCGGATAACAATTTCACACAGGAAACAGCTATGACCATGATTACGCCATGCTATTTAGGTGACACTATAGAATACGCGGCCGCAAGCTTGCATGCCTGCAGGTCGACTCTAGAGCGGATCCACAATTATCGTACGCGTAGTGCTTCGATATCGATCGTTTGGACCCTCCTTCATCACCTATCTTCCTCTCACAGGCCACCCCCCGGATCCATGGTGAGTAAGGGAGAGGAAGATAATATGGCCTCCCTTCCCGCTACGCACGAACTCCACATCTTCGGGTCAATCAACGGTGTTGACTTCGACATGGTGGGCCAGGGCACCGGCAATCCCAATGACGGATACGAAGAACTCAATTTGAAGAGTACAAAGGGCGATCTCCAATTCTCACCTTGGATTCTGGTTCCCCACATTGGATACGGATTTCATCAGTACCTGCCGTACCCCGATGGGATGAGCCCATTTCAGGCTGCAATGGTAGATGGTAGCGGTTACCAAGTACACCGAACTATGCAATTTGAGGACGGTGCCTCACTGACAGTGAACTATCGGTATACTTACGAAGGAAGCCACATCAAGGGAGAGGCACAGGTCAAAGGAACCGGATTTCCAGCCGACGGGCCAGTCATGACAAACTCCCTGACCGCCGCAGATTGGTGCCGCAGCAAAAAGACCTATCCAAATGACAAGACCATTATCTCGACATTCAAATGGAGCTACACCACCGGAAACGGCAAACGCTATCGGTCTACCGCCAGGACAACCTACACATTTGCAAAACCTATGGCCGCAAACTATCTGAAAAACCAGCCGATGTATGTGTTCCGAAAGACGGAATTAAAACACTCGAAAACAGAACTAAACTTTAAAGAGTGGCAGAAAGCCTTTACCGACGTAATGGGCATGGACGAGCTGTATAAGGGAAGCGGAGAGGGCAGAGGAAGTCTGCTAACATGCGGTGACGTCGAGGAGAATCCTGGACCTATGACCGAGTACAAGCCCACGGTGCGCCTCGCCACCCGCGACGACGTCCCCCGGGCCGTACGCACCCTCGCCGCCGCGTTCGCCGACTACCCCGCCACGCGCCACACCGTCGACCCGGACCGCCACATCGAGCGGGTCACCGAGCTGCAAGAACTCTTCCTCACGCGCGTCGGGCTCGACATCGGCAAGGTGTGGGTCGCGGACGACGGCGCCGCGGTGGCGGTCTGGACCACGCCGGAGAGCGTCGAAGCGGGGGCGGTGTTCGCCGAGATCGGCCCGCGCATGGCCGAGTTGAGCGGTTCCCGGCTGGCCGCGCAGCAACAGATGGAAGGCCTCCTGGCGCCGCACCGGCCCAAGGAGCCCGCGTGGTTCCTGGCCACCGTCGGCGTCTCGCCCGACCACCAGGGCAAGGGTCTGGGCAGCGCCGTCGTGCTCCCCGGAGTGGAGGCGGCCGAGCGCGCCGGGGTGCCCGCCTTCCTGGAGACCTCCGCGCCCCGCAACCTCCCCTTCTACGAGCGGCTCGGCTTCACCGTCACCGCCGACGTCGAGGTGCCCGAAGGACCGCGCACCTGGTGCATGACCCGCAAGCCCGGTGCCTGACCAAGGTGAAGAACTGAAGTTCAGCGCTGTCAGGATTGCGCCAAACGATCGATATCGAAGCACTACGCGTACGATAGATCTGGCCTCGAGATCCCGGGTACCGAGCTCGAATTCTGCGGCCGCGCCCTATAGTGAGTCGTATTACACTAGCTCACTGGCCGTCGTTTTACAACGTCGTGACTGGGAAAACCCTGGCGTTACCCAACTTAATCGCCTTGCAGCACATCCCCCTTTCGCCAGCTGGCGTAATAGCGAAGAGGCCCGCACCGATCGCCCTTCCCAACAGTTGCGCAGCCTGAATGGCGAATGGCGCCTGTAGTAATATTTTCTCCTTACGCATCTGTGCGGTATTTCACACCGCATATGGTGCACTCTCAGTACAATCTGCTCTGATGCCGCATAGTTAAGCCAGCCCCGACACCCGCCAACACCCGCTGACGCGCCCTGACGGGCTTGTCTGCTCCCGGCATCCGCTTACAGACAAGCTGTGACCGTCTCCGGGAGCTGCATGTGTCAGAGGTTTTCACCGTCATCACCGAAACGCGCGA

> PITCh(*gRNA-s1*)-*hACTB* (Green: mNeonGreen, Orange: 2A, Blue: Puro)

CTGACGCGCCCTGTAGCGGCGCATTAAGCGCGGCGGGTGTGGTGGTTACGCGCAGCGTGACCGCTACACTTGCCAGCGCCCTAGCGCCCGCTCCTTTCGCTTTCTTCCCTTCCTTTCTCGCCACGTTCGCCGGCTTTCCCCGTCAAGCTCTAAATCGGGGGCTCCCTTTAGGGTTCCGATTTAGTGCTTTACGGCACCTCGACCCCAAAAAACTTGATTAGGGTGATGGTTCACGTAGTGGGCCATCGCCCTGATAGACGGTTTTTCGCCCTTTGACGTTGGAGTCCACGTTCTTTAATAGTGGACTCTTGTTCCAAACTGGAACAACACTCAACCCTATCTCGGTCTATTCTTTTGATTTATAAGGGATTTTGCCGATTTCGGCCTATTGGTTAAAAAATGAGCTGATTTAACAAAAATTTAACGCGAATTTTAACAAAATATTAACGCTTACAATTTCCATTCGCCATTCAGGCTGCGCAACTGTTGGGAAGGGCGATCGGTGCGGGCCTCTTCGCTATTACGCCAGCTGGCGAAAGGGGGATGTGCTGCAAGGCGATTAAGTTGGGTAACGCCAGGGTTTTCCCAGTCACGACGTTGTAAAACGACGGCCAGTGAGCGCGCGTAATACGACTCACTATAGGGCGAATTGGGTACCGGGCCCCCCCTCGAGGTCGACGGTATCGATAAGCTTGATATCGAATTCCCAATACTATCGTACGCGTAGTGCTTCGATATCGATCGTTTGGTATGACGAGTCCGGCCCCTCCATCGTCCACCGCAAATGCTTCGGATCCATGGTGAGTAAGGGAGAGGAAGATAATATGGCCTCCCTTCCCGCTACGCACGAACTCCACATCTTCGGGTCAATCAACGGTGTTGACTTCGACATGGTGGGCCAGGGCACCGGCAATCCCAATGACGGATACGAAGAACTCAATTTGAAGAGTACAAAGGGCGATCTCCAATTCTCACCTTGGATTCTGGTTCCCCACATTGGATACGGATTTCATCAGTACCTGCCGTACCCCGATGGGATGAGCCCATTTCAGGCTGCAATGGTAGATGGTAGCGGTTACCAAGTACACCGAACTATGCAATTTGAGGACGGTGCCTCACTGACAGTGAACTATCGGTATACTTACGAAGGAAGCCACATCAAGGGAGAGGCACAGGTCAAAGGAACCGGATTTCCAGCCGACGGGCCAGTCATGACAAACTCCCTGACCGCCGCAGATTGGTGCCGCAGCAAAAAGACCTATCCAAATGACAAGACCATTATCTCGACATTCAAATGGAGCTACACCACCGGAAACGGCAAACGCTATCGGTCTACCGCCAGGACAACCTACACATTTGCAAAACCTATGGCCGCAAACTATCTGAAAAACCAGCCGATGTATGTGTTCCGAAAGACGGAATTAAAACACTCGAAAACAGAACTAAACTTTAAAGAGTGGCAGAAAGCCTTTACCGACGTAATGGGCATGGACGAGCTGTATAAGGGAAGCGGAGAGGGCAGAGGAAGTCTGCTAACATGCGGTGACGTCGAGGAGAATCCTGGACCTATGACCGAGTACAAGCCCACGGTGCGCCTCGCCACCCGCGACGACGTCCCCCGGGCCGTACGCACCCTCGCCGCCGCGTTCGCCGACTACCCCGCCACGCGCCACACCGTCGACCCGGACCGCCACATCGAGCGGGTCACCGAGCTGCAAGAACTCTTCCTCACGCGCGTCGGGCTCGACATCGGCAAGGTGTGGGTCGCGGACGACGGCGCCGCGGTGGCGGTCTGGACCACGCCGGAGAGCGTCGAAGCGGGGGCGGTGTTCGCCGAGATCGGCCCGCGCATGGCCGAGTTGAGCGGTTCCCGGCTGGCCGCGCAGCAACAGATGGAAGGCCTCCTGGCGCCGCACCGGCCCAAGGAGCCCGCGTGGTTCCTGGCCACCGTCGGCGTCTCGCCCGACCACCAGGGCAAGGGTCTGGGCAGCGCCGTCGTGCTCCCCGGAGTGGAGGCGGCCGAGCGCGCCGGGGTGCCCGCCTTCCTGGAGACCTCCGCGCCCCGCAACCTCCCCTTCTACGAGCGGCTCGGCTTCACCGTCACCGCCGACGTCGAGGTGCCCGAAGGACCGCGCACCTGGTGCATGACCCGCAAGCCCGGTGCCTGATCTAGGCGGACTATGACTTAGTTGCGTTACACCCTTTCTTCCAAACGATCGATATCGAAGCACTACGCGTACGATAGTATTGGGAATTCCTGCAGCCCGGGGGATCCACTAGTTCTAGAGCGGCCGCCACCGCGGTGGAGCTCCAGCTTTTGTTCCCTTTAGTGAGGGTTAATTGCGCGCTTGGCGTAATCATGGTCATAGCTGTTTCCTGTGTGAAATTGTTATCCGCTCACAATTCCACACAACATACGAGCCGGAAGCATAAAGTGTAAAGCCTGGGGTGCCTAATGAGTGAGCTAACTCACATTAATTGCGTTGCGCTCACTGCCCGCTTTCCAGTCGGGAAACCTGTCGTGCCAGCTGCATTAATGAATCGGCCAACGCGCGGGGAGAGGCGGTTTGCGTATTGGGCGCTCTTCCGCTTCCTCGCTCACTGACTCGCTGCGCTCGGTCGTTCGGCTGCGGCGAGCGGTATCAGCTCACTCAAAGGCGGTAATACGGTTATCCACAGAATCAGGGGATAACGCAGGAAAGAACATGTGAGCAAAAGGCCAGCAAAAGGCCAGGAACCGTAAAAAGGCCGCGTTGCTGGCGTTTTTCCATAGGCTCCGCCCCCCTGACGAGCATCACAAAAATCGACGCTCAAGTCAGAGGTGGCGAAACCCGACAGGACTATAAAGATACCAGGCGTTTCCCCCTGGAAGCTCCCTCGTGCGCTCTCCTGTTCCGACCCTGCCGCTTACCGGATACCTGTCCGCCTTTCTCCCTTCGGGAAGCGTGGCGCTTTCTCATAGCTCACGCTGTAGGTATCTCAGTTCGGTGTAGGTCGTTCGCTCCAAGCTGGGCTGTGTGCACGAACCCCCCGTTCAGCCCGACCGCTGCGCCTTATCCGGTAACTATCGTCTTGAGTCCAACCCGGTAAGACACGACTTATCGCCACTGGCAGCAGCCACTGGTAACAGGATTAGCAGAGCGAGGTATGTAGGCGGTGCTACAGAGTTCTTGAAGTGGTGGCCTAACTACGGCTACACTAGAAGGACAGTATTTGGTATCTGCGCTCTGCTGAAGCCAGTTACCTTCGGAAAAAGAGTTGGTAGCTCTTGATCCGGCAAACAAACCACCGCTGGTAGCGGTGGTTTTTTTGTTTGCAAGCAGCAGATTACGCGCAGAAAAAAAGGATCTCAAGAAGATCCTTTGATCTTTTCTACGGGGTCTGACGCTCAGTGGAACGAAAACTCACGTTAAGGGATTTTGGTCATGAGATTATCAAAAAGGATCTTCACCTAGATCCTTTTAAATTAAAAATGAAGTTTTAAATCAATCTAAAGTATATATGAGTAAACTTGGTCTGACAGTTACCAATGCTTAATCAGTGAGGCACCTATCTCAGCGATCTGTCTATTTCGTTCATCCATAGTTGCCTGACTCCCCGTCGTGTAGATAACTACGATACGGGAGGGCTTACCATCTGGCCCCAGTGCTGCAATGATACCGCGAGACCCACGCTCACCGGCTCCAGATTTATCAGCAATAAACCAGCCAGCCGGAAGGGCCGAGCGCAGAAGTGGTCCTGCAACTTTATCCGCCTCCATCCAGTCTATTAATTGTTGCCGGGAAGCTAGAGTAAGTAGTTCGCCAGTTAATAGTTTGCGCAACGTTGTTGCCATTGCTACAGGCATCGTGGTGTCACGCTCGTCGTTTGGTATGGCTTCATTCAGCTCCGGTTCCCAACGATCAAGGCGAGTTACATGATCCCCCATGTTGTGCAAAAAAGCGGTTAGCTCCTTCGGTCCTCCGATCGTTGTCAGAAGTAAGTTGGCCGCAGTGTTATCACTCATGGTTATGGCAGCACTGCATAATTCTCTTACTGTCATGCCATCCGTAAGATGCTTTTCTGTGACTGGTGAGTACTCAACCAAGTCATTCTGAGAATAGTGTATGCGGCGACCGAGTTGCTCTTGCCCGGCGTCAATACGGGATAATACCGCGCCACATAGCAGAACTTTAAAAGTGCTCATCATTGGAAAACGTTCTTCGGGGCGAAAACTCTCAAGGATCTTACCGCTGTTGAGATCCAGTTCGATGTAACCCACTCGTGCACCCAACTGATCTTCAGCATCTTTTACTTTCACCAGCGTTTCTGGGTGAGCAAAAACAGGAAGGCAAAATGCCGCAAAAAAGGGAATAAGGGCGACACGGAAATGTTGAATACTCATACTCTTCCTTTTTCAATATTATTGAAGCATTTATCAGGGTTATTGTCTCATGAGCGGATACATATTTGAATGTATTTAGAAAAATAAACAAATAGGGGTTCCGCGCACATTTCCCCGAAAAGTGCCAC

**Figure S17** The full plasmid sequences of MMEJ reporter vector, PITCh(*gRNA-s1*)-*FBL* donor vector, and PITCh(*gRNA-s1*)-*hACTB* donor vector.

| **Locus** | **Score** | **Sequence** | **Mismatch** | **Chromosome position** | **Gene** | **Allele type (5’)*** | **Allele type (3’)*** |
| --- | --- | --- | --- | --- | --- | --- | --- |
| On-target | 0 | CGTCCACCGCAAATGCTTCTAGG – hit  CGTCCACCGCAAATGCTTCTNGG - query | 0 | Chr7:5527747-5527769 | ACTB (actin, beta) | - | - |
| OT1 | 0 | CGTCCACCGCAAATGCTTCTAGG - hit  CGTCCACCGCAAATGCTTCTNGG - query | 0 | Chr5:80299677-80299699 | LOC644936 (actin, beta pseudogene) | - | III |
| OT2 | 0.12 | TGTCCACCGCAAATGCTTCTAGG – hit  CGTCCACCGCAAATGCTTCTNGG - query | 1 | Chr2:130657856-130657878 | POTEJ (POTE ankyrin domain family member J) | I | - |
| OT3 | 0.12 | TGTCCACCGCAAATGCTTCTAGG - hit  CGTCCACCGCAAATGCTTCTNGG - query | 1 | Chr2:131264662-131264684 | POTEE (POTE ankyrin domain family member E) | I | - |
| OT4 | 0.12 | TGTCCACCGCAAATGCTTCTAGG - hit  CGTCCACCGCAAATGCTTCTNGG - query | 1 | Chr2:131627245-131627267 | Intergenic | I | - |
| OT5 | 0.12 | TGTCCACCGCAAATGCTTCTAGG - hit  CGTCCACCGCAAATGCTTCTNGG - query | 1 | Chr14:18999974-18999996 | POTEM (POTE ankyrin domain family, member M), ACTBP10 (actin, beta pseudogene 10) | I | IV |
| OT6 | 0.12 | TGTCCACCGCAAATGCTTCTAGG - hit  CGTCCACCGCAAATGCTTCTNGG - query | 1 | Chr22:15722590-15722612 | Intergenic | - | IV |
| OT7 | 0.12 | TGTCCACCGCAAATGCTTCTAGG - hit  CGTCCACCGCAAATGCTTCTNGG - query | 1 | Chr2:130462815-130462837 | POTEI (POTE ankyrin domain family member I) | I | - |
| OT8 | 0.12 | TGTCCACCGCAAATGCTTCTAGG - hit  CGTCCACCGCAAATGCTTCTNGG - query | 1 | Chr14:19401780-19401802 | Intergenic | I | IV |
| OT9 | 0.13 | CCTCCACCGCAAATGCTTCTAGG - hit  CGTCCACCGCAAATGCTTCTNGG - query | 1 | Chr11:1802888-1802910 | LOC390029 (actin, beta pseudogene) | - | - |
| OT10 | 0.17 | CGTTCACCGCAAATGCTTCTAGG - hit  CGTCCACCGCAAATGCTTCTNGG - query | 1 | Chr5:77785987-77786009 | ACTBP2 (actin, beta pseudogene 2) | - | - |
| OT11 | 0.23 | CGTCCATCGCAAATGCTTCTAGG - hit  CGTCCACCGCAAATGCTTCTNGG - query | 1 | Chr5:131659045-131659067 | FNIP1 (folliculin interacting protein 1), ACTBP4 (actin, beta pseudogene 4) | - | - |
| OT12 | 0.35 | CGTCCACCACAAATGCTTCTAGG - hit  CGTCCACCGCAAATGCTTCTNGG - query | 1 | Chr18:62443108-62443130 | ACTBP9 (actin, beta pseudogene 9) | - | - |
| OT13 | 0.35 | CGTCCACCACAAATGCTTCTAGG - hit  CGTCCACCGCAAATGCTTCTNGG - query | 1 | Chr6:88275881-88275903 | ACTBP8 (actin, beta pseudogene 8) | - | - |
| OT14 | 0.39 | TGTCCACTGCAAATGCTTCTAGG - hit  CGTCCACCGCAAATGCTTCTNGG - query | 2 | Chr3:175977492-175977514 | ACTG1P23 (actin gamma 1 pseudogene 23) | II | - |

*Four kinds of allele types were detected by DNA sequencing, related to **Figure 4d, 4e, 5a, S15, and S16**.

**Table S1.** The on-target and potential off-target sites of sgRNA targeting *hACTB* gene. The top fourteen off-target sites according to the scores calculated by the COSMID software are shown with the on-target site. Red letters indicate mismatched bases.

**Table S2** Mutations at putative off-target loci containing up to 3 bp mismatches for *Actb* target are analyzed by sequencing. Mismatches compared to on-target sequence are shown in red. PAM sequences are labeled in blue.

| **Line** | **Exo1** | **Sex** | **Mating Strain** | **F1 Newborn** | **F1 Knockin (%)** |
| --- | --- | --- | --- | --- | --- |
| KI#1 | - | Female | BDF1 | 24 | 12 (50.0) |
| KI#2 | - | Female | C57BL/6 | 24 | 8 (33.3) |
| KI#3 | - | Female | C57BL/6 | 9 | 6 (66.7) |
| eKI#1 | + | Male | C57BL/6 | 36 | 12 (33.3) |
| eKI#3 | + | Female | BDF1 | 19 | 13 (68.4) |
| eKI#4 | + | Male | C57BL/6 | 15 | 8 (53.3) |
| eKI#5 | + | Male | C57BL/6 | 14 | 7 (50.0) |
| Total |  |  |  | 141 | 66 (46.8) |

**Table S3** Germline transmission of knockin alleles. Percentages were calculated using the number in each column as the numerator and the number in the column to its left as the denominator.

| sgRNA cloning | | |
| --- | --- | --- |
| Name | Direction | Sequence (5’ to 3’) |
| MMEJ reporter | F | CACCGCACGCCGTAGGTCAGTCGT |
|  | R | AAACACGACTGACCTACGGCGTGC |
| FBL | F | CACCGCTCTCACAGGCCACCCCCCA |
|  | R | AAACTGGGGGGTGGCCTGTGAGAGC |
| hACTB | F | CACCGCGTCCACCGCAAATGCTTCT |
|  | R | AAACAGAAGCATTTGCGGTGGACGC |
| gRNA-s1 | F | CACCGTGCTTCGATATCGATCGTT |
|  | R | AAACAACGATCGATATCGAAGCAC |

| Amplification of knock-in junctions in human cell experiments | | |
| --- | --- | --- |
| Name | Direction | Sequence (5’ to 3’) |
| 5’ junction of FBL | F | ACACCAAGACAGACATCTCTGTCCCTTG |
|  | R | ATCCGTATCCAATGTGGGGAAC |
| 3’ junction of FBL | F | CACCAGGGCAAGGGTCTG |
|  | R | TCAGCAGGTCAAGGGGAGGAATG |
| 5’ junction of hACTB | F | GAGCGCAAGTACTCCGTGTGGATC |
|  | R | ATCCGTATCCAATGTGGGGAAC |
| 3’ junction of hACTB | F | CACCAGGGCAAGGGTCTG |
|  | R | TCACCTTCACCGTTCCAGTTTTTAAATCC |

| Overexpression vector construction | | |
| --- | --- | --- |
| Name | Direction | Sequence (5’ to 3’) |
| Exo1-vector | F | AGAGCAATATTCCAGTAAAAAATCAGCCTCGACTGTGCCTTC |
|  | R | TCCCTGTATCCCCATGGTGGCCTCGAGGCC |
| Exo1-insert | F | ATGGGGATACAGGGATTGCTACAATTTATC |
|  | R | CTGGAATATTGCTCTTTGAACACGGC |
| Lig3-vector | F | GTAGCTCCCTGCTAGAAAATCAGCCTCGACTGTGCCTTC |
|  | R | GAAAGCCAAAGACATGGTGGCCTCGAGGCC |
| Lig3-insert | F | ATGTCTTTGGCTTTCAAGATCTTCTTTCC |
|  | R | CTAGCAGGGAGCTACCAGTCTCCG |
| PARP1-vector | F | ACCTCCCTGTGGTAAAAAATCAGCCTCGACTGTGCCTTC |
|  | R | CGAAGACTCCGCCATGGTGGCCTCGAGGCC |
| PARP1-insert | F | ATGGCGGAGTCTTCGGATAAGCTC |
|  | R | TTACCACAGGGAGGTCTTAAAATTGAATTTC |
| NBS1-vector | F | AAAAGGAGAAGATAAAAAATCAGCCTCGACTGTGCCTTC |
|  | R | CAGCAGTTTCCACATGGTGGCCTCGAGGCC |
| NBS1-insert | F | ATGTGGAAACTGCTGCCC |
|  | R | TTATCTTCTCCTTTTTAAATAAGGATTG |
| FEN1-vector | F | AAAAGGGGAAAATAAAAAATCAGCCTCGACTGTGCCTTC |
|  | R | GCCTTGAATTCCCATGGTGGCCTCGAGGCC |
| FEN1-insert | F | ATGGGAATTCAAGGCCTGGCC |
|  | R | TTATTTTCCCCTTTTAAACTTCCCTGCTG |
| BLM-vector | F | TATGCATTCTCATAAAAAATCAGCCTCGACTGTGCCTTC |
|  | R | AGGAACAGCAGCCATGGTGGCCTCGAGGCC |
| BLM-insert1 | F | ATGGCTGCTGTTCCTCAAAATAATCTACAG |
|  | R | GATAGGCAGCTGTGGAAGATTTGCTG |
| BLM-insert2 | F | CCACAGCTGCCTATCAACCCATC |
|  | R | TTATGAGAATGCATATGAAGGCTTAAGAAACG |
| MRE11A-vector | F | AGAAGAAATAGAAGATAAAAAATCAGCCTCGACTGTGCCTTC |
|  | R | ATCTGCAGTACTCATGGTGGCCTCGAGGCC |
| MRE11A-insert | F | ATGAGTACTGCAGATGCACTTG |
|  | R | TCTTCTATTTCTTCTTAAAGAACTAGTGTTC |
| TREX2-vector | F | CCCAGCCTGGAGGCCTAAAAAATCAGCCTCGACTGTGCCTTC |
|  | R | TCCGAGGCACCCCATGGTGGCCTCGAGGCC |
| TREX2-insert | F | ATGTCCGAGGCACCCCGG |
|  | R | GGCCTCCAGGCTGGGGTC |
| RAD51-vector | F | GGAGATGCCAAAGACTAAAAAATCAGCCTCGACTGTGCCTTC |
|  | R | CATCTGCATTGCCATGGTGGCCTCGAGGCC |
| RAD51-insert | F | ATGGCAATGCAGATGCAGCTTG |
|  | R | GTCTTTGGCATCTCCCACTCCATC |
| RAD52-vector | F | AAATATGATCCATCTTAAAAAATCAGCCTCGACTGTGCCTTC |
|  | R | CTCAGTCCCAGACATGGTGGCCTCGAGGCC |
| RAD52-insert | F | ATGTCTGGGACTGAGGAAGCAATTCTTG |
|  | R | AGATGGATCATATTTCCTTTTCTTCATGTCCTG |
| DN-Lig4-vector | F | AACCAGTATTTGATTTAAAAAATCAGCCTCGACTGTGCC |
|  | R | ATTAGAAATTTTGTTCATGGTGGCCTCGAGGCC |
| DN-Lig4-insert | F | AACAAAATTTCTAATATATTTGAAGATG |
|  | R | AATCAAATACTGGTTTTCTTCTTG |
| DN-Ku70-vector | F | AAGCACTTCCAGGACTAAAAAATCAGCCTCGACTGTGCC |
|  | R | GATACACTGGATGCTCATGGTGGCCTCGAGGCC |
| DN-Ku70-insert | F | ATGAGCATCCAGTGTATCCAAAGTGTG |
|  | R | GTCCTGGAAGTGCTTGGTGAGG |

| crRNAs and tracrRNA | |
| --- | --- |
| Name | Sequence (5’ to 3’) |
| Actb-crRNA | cauuaugaguccuuaagugaGUUUUAGAGCUAUGCUGUUUUG |
| Col12a1-left-crRNA | ugacuuccaugguuccacaaGUUUUAGAGCUAUGCUGUUUUG |
| Col12a1-right-crRNA | cacagcacuguacagaauagGUUUUAGAGCUAUGCUGUUUUG |
| gRNA-s1-crRNA | gugcuucgauaucgaucguuGUUUUAGAGCUAUGCUGUUUUG |
| tracrRNA | AAACAGCAUAGCAAGUUAAAAUAAGGCUAGUCCGUUAUCAACUUGAAAAAGUGGCACCGAGUCGGUGCU |

| Genotyping | | |
| --- | --- | --- |
| Name | Direction | Sequence (5’ to 3’) |
| LF | F | TGCAGAGAACACTGGTTGGT |
| LR | R | ATCCCAATTCTTTGCCAAAGTG |
| RF | F | GGACTTTCGCTTTCCCCC |
| RR | R | CAAGCTAACCTCAGCCTTGC |
| IF | F | ATGTAGCACAGCCAGTAGCC |
| IR | R | CTCCGACGAGGAGGACATTG |
| LF (col12a1) | F | GCAGTATGAAGTCATGTGCGG |
| RR (col12a1) | R | AAGGTCACAGTCCTGACCCA |

| Off-target analysis | | |
| --- | --- | --- |
| Name | Direction | Sequence (5’ to 3’) |
| OT1 | F | ATGCCCAGGAGTGGTGTTAC |
|  | R | TCTTGGGCCATCTTCATAGC |
| OT2 | F | TCCGTAGCTAGCAAGGAGAGT |
|  | R | AGTGTTACTGGTGTTTGATGGGT |
| OT3 | F | TCTTGGTTCCTTTATTTCTCAGAGG |
|  | R | AGTGTTGGATCTCTGGTCGC |
| OT4 | F | GTGCTGCCCTAGGGGATATT |
|  | R | CTCACTCCCATCTTTGTGGGG |
| OT5 | F | ACCTTAAACACATGTTCTGCCAC |
|  | R | ATACCACCTGAAGGTTGGGG |

**Table S4** Oligo DNAs, primers, and RNAs used in this study.
